# Single-cell and bulk transcriptomic analyses reveal PANoptosis-associated immune dysregulation of fibroblasts in periodontitis

**DOI:** 10.3389/fimmu.2025.1671919

**Published:** 2025-09-05

**Authors:** Erli Wu, Qiangqiang Zhuo, Xuan Yin, Jingjing Li, Huijuan Zhang, Feihan Gu, Feng Liang, Xianqing Zhou, Ziyang Gao, Bang Li, Qingqing Wang, Wei Shao

**Affiliations:** ^1^ College & Hospital of Stomatology, Anhui Medical University, Key Lab. of Oral Diseases Research of Anhui, Hefei, China; ^2^ Department of Periodontology, Anhui Stomatology Hospital affiliated to Anhui Medical University, Hefei, China; ^3^ Department of Microbiology and Parasitology, Anhui Provincial Laboratory of Pathogen Biology, School of Basic Medical Sciences, Anhui Medical University, Hefei, Anhui, China

**Keywords:** panoptosis, periodontitis, fibroblasts, immune dysregulation, spatial transcriptome, machine learning

## Abstract

**Background:**

PANoptosis is a newly recognized form of programmed inflammatory cell death implicated in numerous inflammation-related diseases. However, its precise role and underlying mechanisms in periodontitis (PD) remain unclear.

**Methods:**

We analyzed single-cell RNA sequencing (scRNA-seq) on gingival tissues from PD patients and healthy individuals to profile cellular composition and quantify cell-type distributions. Functional enrichment analyses were used to explore PANoptosis and related pathways, with five gene set scoring methods applied to quantify PANoptosis activity in human gingival fibroblasts (HGFs). The expression of PANoptosis-related markers was validated by immunofluorescence staining and qPCR in HGFs and gingival tissues from PD model mice. Based on PANoptosis scores, HGFs were stratified into high- and low-activity groups. Cell-cell communication and spatial transcriptomic analyses were integrated to examine their interactions with immune cells in the periodontal microenvironment. Finally, bulk RNA-seq data were subjected to comprehensive analysis using 113 machine learning models to screen for core PANoptosis-associated genes, which were subsequently validated through qPCR and immunohistochemistry in gingival tissues.

**Results:**

scRNA-seq analysis revealed a decreased proportion of HGFs alongside enrichment of multiple PANoptosis-related pathways in PD samples. Further assessment demonstrated significantly elevated PANoptosis activity in HGFs from PD compared to controls, which was validated by tissue-level immunofluorescence staining. *In vitro* experiments using cultured HGFs and *in vivo* analyses in PD model mice further confirmed upregulation of PANoptosis-related markers via immunofluorescence and qPCR. Upon stratifying HGFs into high- and low-PANoptosis groups, cell-cell communication and spatial transcriptomic analyses indicated that high-PANoptosis HGFs exhibited enhanced interactions with immune cells within the periodontal microenvironment. Additionally, bulk transcriptomic profiling combined with machine learning approaches identified four key PANoptosis-associated genes, which were subsequently validated in human gingival tissues.

**Conclusion:**

Our findings demonstrate that PANoptosis is activated in HGFs in the context of PD, which may drive immune dysregulation and facilitate disease progression. By integrating bulk transcriptomic data with machine learning algorithms, we identified and validated key PANoptosis-related genes, highlighting their potential as novel therapeutic targets.

## 1 Introduction

Periodontitis (PD) is a widespread chronic inflammatory disease, affecting more than 40% of adults globally and standing as a primary cause of tooth loss among the elderly (1). The onset of PD involves a dysbiotic shift in the oral microbiome, which provokes an excessive host immune response marked by elevated levels of pro-inflammatory cytokines (such as TNF-α and IL-1β) and matrix metalloproteinases (MMPs), ultimately resulting in the breakdown of the periodontal ligament and alveolar bone (2). Although the central role of inflammation in PD pathogenesis is well established, the precise cellular and molecular mechanisms underlying irreversible tissue damage remain incompletely understood.

Recent studies have highlighted the importance of programmed cell death (PCD) in shaping the periodontal microenvironment (3). Among the known PCD pathways, apoptosis, pyroptosis and necroptosis have been extensively implicated in PD, especially in the context of immune activation and microbial insult (4, 5). However, emerging evidence suggests that these forms of cell death often do not occur in isolation, but instead act in a coordinated and context-dependent manner (6). PANoptosis, a recently defined and mechanistically integrated cell death pathway, represents a novel paradigm that unifies pyroptosis, apoptosis, and necroptosis into a single, tightly regulated inflammatory program (7). Unlike the individual pathways, PANoptosis is triggered by specific upstream signals—such as ZBP1, AIM2, or TNF—and is executed through the assembly of PANoptosomes, leading to the simultaneous activation of multiple PCD effectors (e.g., CASP1, CASP8, RIPK3) (8, 9). This results in rapid, lytic cell death accompanied by robust release of DAMPs, thereby amplifying inflammation.

Recent studies have implicated PANoptosis in acute infections, sepsis, autoimmune diseases and other chronic inflammatory conditions, where it mediates inflammatory cell death and amplifies immune responses (10–13). However, its potential role in PD remains largely unexplored. The periodontal environment—with its persistent bacterial challenge, oxidative stress, and cytokine-rich milieu—provides an ideal setting for PANoptotic activation. Importantly, PANoptosis is not merely redundant with pyroptosis or necroptosis, but may serve as a higher-order regulatory mechanism that determines cell fate under complex inflammatory stimuli (14). Its activation could explain cases where multiple PCD markers are simultaneously upregulated, as has been observed in transcriptomic studies of periodontal lesions (15, 16). Meanwhile, Jiang et al. further investigated the relationship between cell death induced by *Porphyromonas gingivalis* (*P. gingivalis*)—a keystone periodontal pathogen—and PD progression, proposing that PANoptosis may facilitate immune evasion by *P. gingivalis*, thereby acting as a potential pathogenic mechanism in PD (17). Human gingival fibroblasts (HGFs)—a major cellular constituent of gingival connective tissue—are increasingly recognized as active participants in PD pathogenesis rather than passive structural cells (18). HGFs respond to microbial stimuli by secreting pro-inflammatory cytokines and chemokines, and are known to undergo apoptosis, pyroptosis, and necroptosis upon infection with *P. gingivalis* (19–21). Given this susceptibility and their functional heterogeneity, HGFs may undergo PANoptotic reprogramming that shapes the local immune microenvironment and disease progression. Therefore, identifying PANoptosis-stage-specific HGF subtypes is therefore critical to understanding how this integrated cell death mechanism contributes to periodontal tissue destruction.

In this study, we leveraged publicly available single-cell RNA sequencing (scRNA-seq) datasets to profile PANoptosis activity in HGFs from healthy and diseased gingival tissues. Through integrative analyses including cell–cell communication, spatial transcriptomics, and machine learning, we uncovered PANoptosis-associated HGF subsets, mapped their spatial niches and intercellular interactions, and identified diagnostic biomarkers with translational relevance. These findings provide new insights into PANoptosis as a central pathological mechanism in PD and offer a foundation for developing targeted therapeutic strategies.

## 2 Materials and methods

### 2.1 Data collection

We obtained the single-cell RNA-seq dataset GSE164241 and bulk RNA-seq datasets GSE16134 and GSE10334 from the GEO database (https://www.ncbi.nlm.nih.gov/geo/) for integrated analysis. The single-cell dataset included 21 gingival tissue samples, with 13 from healthy controls and 8 from PD patients. For bulk transcriptomic analysis, we employed two complementary GEO datasets: GSE16134, comprising 310 samples (241 PD and 69 healthy controls) as the training set, and GSE10334, including 247 samples (183 PD and 64 controls) as the independent validation cohort. Additionally, to explore the spatial distribution and localization of cells within PD-affected gingival tissues, we incorporated two PD samples (GSM6258258 and GSM6258257) from the spatial transcriptomic dataset GSE206621. This spatial transcriptomics data enabled us to investigate the *in situ* cellular architecture and spatial relationships of key cell populations identified in the single-cell analysis, providing critical spatial context to complement the molecular findings. **Supplementary Tables 1–4** listed the genes related to PANoptosis, apoptosis, pyroptosis, and necroptosis used in this study.

### 2.2 scRNA-seq data processing

To identify cell populations associated with PANoptosis and characterize their transcriptional profiles, we employed Seurat (v4.3.1) in R for comprehensive single-cell analysis. Cells expressing between 200 and 6,000 genes and with mitochondrial content under 20% passed quality control. After normalization, the top 2,000 highly variable genes were selected. Data scaling and principal component analysis (PCA) were performed, followed by batch correction and integration using Harmony (v1.2.3). For visualization and clustering, UMAP was applied on the top 20 PCs. Cluster markers were identified with FindAllMarkers, using a log fold change > 0.25 and minimum expression in 25% of cells. Cell clusters were subsequently annotated according to canonical cell surface markers and curated reference datasets from previously published literature.

### 2.3 PANoptosis-related gene set activity scoring analysis

To evaluate PANoptosis activity in HGFs, we curated a gene set of 109 PANoptosis-related genes. We assessed their activity at the single-cell level using five distinct gene set scoring algorithms: AUCell, UCell, singscore, ssGSEA, and AddModuleScore. Specifically, AUCell (v1.24.0) was used to build cell-wise gene expression rankings and calculate area under the curve (AUC) values for the PANoptosis gene set. UCell and singscore were applied using the irGSEA package (v3.3.2) to generate normalized scores based on non-parametric and rank-based methods. ssGSEA computed enrichment scores across all single cells using a Gaussian kernel. AddModuleScore calculated average expression levels of the PANoptosis gene set after control gene subtraction. To integrate and compare results across methods, all individual scores were min-max normalized (0–1 range). A composite PANoptosis activity score was then calculated by summing the normalized scores from all five algorithms for each cell, providing a robust and comprehensive measurement of PANoptotic activity. We utilized the “ggplot2” R package (v3.5.1) to visualize gene set scores at the single-cell level, enabling comparison of pathway activities in HGFs from both healthy and PD samples. The same scoring framework was also applied to assess the activity of apoptosis, pyroptosis, and necroptosis pathways, using curated gene sets specific to each cell death program.

### 2.4 Pseudotime trajectory analysis

Using the “Monocle 2” R package (v2.30.1), we conducted pseudotime analysis to investigate the developmental progression of HGFs subpopulations exhibiting varying levels of PANoptosis activity. Cells were ordered along a pseudotime axis based on highly variable genes using the DDRTree algorithm. Gene expression dynamics and functional pathway changes were analyzed along the trajectory to reveal transcriptional reprogramming associated with PANoptosis during HGFs state transitions.

### 2.5 Cell-cell communication analysis

To investigate the intercellular interactions between cell types in PD, we employed the “CellChat” package (v2.2.0) to infer and visualize cell-cell communication networks (22). The normalized single-cell transcriptomic data were used to construct ligand-receptor interaction networks. The communication strength was quantified by analyzing the overall interaction number and signaling strength.

### 2.6 Functional and pathway enrichment analysis

To explore the biological functions and signaling pathways linked to PANoptosis in PD, we conducted Gene Set Variation Analysis (GSVA) and GSEA to assess functional differences between PANoptosis-related cell subsets. Gene sets were sourced from the Molecular Signatures Database (MSigDB) to ensure comprehensive pathway coverage. Additionally, differentially expressed genes (DEGs) between these subgroups were analyzed for Gene Ontology (GO) and Kyoto Encyclopedia of Genes and Genomes (KEGG) pathway enrichment using the “clusterProfiler” R package (v4.15.1).

### 2.7 Spatial transcriptomics and spatial trajectory analysis

We performed comprehensive ST analysis using “Seurat” in R to process Visium-derived gene-spot matrices, filtering out spots with fewer than 10 detected genes and normalizing the data with SCTransform while accounting for mitochondrial and ribosomal gene effects. Dimensionality reduction and clustering were applied to identify spatial gene expression patterns, followed by visualization using UMAP. For cell type deconvolution, we employed the Robust Cell Type Decomposition (RCT) algorithm to accurately estimate the cellular composition of each spatial spot, offering enhanced robustness against technical noise and compositional variability. To further investigate cell-cell communication and spatial organization within the PD microenvironment, we utilized mistyR (v1.10.0), a multiview machine learning framework that integrates gene expression data with spatial coordinates. This approach enabled quantification of context-specific regulatory influences exerted by neighboring cells and identification of spatially structured intercellular signaling networks, providing deeper insights into the local regulatory architecture driving disease progression.

### 2.8 Consensus clustering of PANoptosis genes

To explore the role of PANoptosis in PD, we performed consensus clustering using the “ConsensusClusterPlus” R package (v1.66.0) to classify patients based on the expression patterns of PANoptosis-related genes (PRGs). The optimal cluster number was identified by evaluating cumulative distribution function (CDF) curves across different clustering iterations. The stability and accuracy of the resulting clusters were further confirmed through PCA.

### 2.9 Immune cell infiltration and functional analysis

To evaluate immune characteristics across the identified subtypes, we applied single-sample Gene Set Enrichment Analysis (ssGSEA) using the “GSVA” R package (v1.50.5). This method quantified the infiltration levels of 28 immune cell types and assessed immune-related functional pathways based on established gene signatures. Differences in immune cell infiltration and pathway activity between subtypes were statistically evaluated using the Wilcoxon rank-sum test and visualized with boxplots. Furthermore, Spearman correlation analysis was conducted to explore relationships between PANoptosis-related gene expression and immune features.

### 2.10 Weighted gene co-expression network analysis

To systematically identify gene modules linked to PANoptosis-related expression patterns, we conducted WGCNA using the “WGCNA” R package (v1.73) (23). An optimal soft-thresholding power was chosen to achieve scale-free network topology, balancing network connectivity with biological significance. Genes were clustered according to topological overlap, and modules were detected via a dynamic tree-cutting algorithm. Each module’s expression pattern was summarized by its module eigengene (ME), defined as the first principal component representing the module’s overall gene expression profile. Correlations between module eigengenes and PANoptosis-related traits or sample phenotypes were then calculated to identify biologically relevant modules. The module showing the strongest correlation was selected for downstream analysis.

### 2.11 Machine learning algorithms

To develop the diagnostic model, we applied a comprehensive machine learning framework incorporating multiple established algorithms to systematically perform feature selection and model training. A total of 113 model variants were generated and evaluated using tenfold cross-validation on the GSE16134 dataset. The performance of each model was further validated in an independent external cohort (GSE10334). The final diagnostic model was selected based on the highest average area under the receiver operating characteristic curve (AUC) across both training and validation datasets.

### 2.12 Profiling and visualization of hub gene expression

Differential expression of key signature genes was analyzed using the R package “limma” (v3.62.2), with visualization performed via boxplots created by the “ggpubr” package (v0.6.0). To assess the diagnostic potential of each core gene, Receiver Operating Characteristic (ROC) curve analysis was conducted using the “pROC” package (v1.18.0), and the AUC values were calculated accordingly.

### 2.13 Tissue sample collection

Gingival tissue samples were collected from 20 individuals at the Stomatology Hospital of Anhui Medical University, including 10 healthy controls and 10 patients with PD. PD was diagnosed based on a probing depth ≥5 mm and clinical attachment loss ≥3 mm in at least two teeth, accompanied by bleeding on probing. Healthy controls had a probing depth ≤3 mm, no clinical attachment loss, and no bleeding on probing; gingival tissues were obtained from the excised gingiva during third molar extraction performed due to orthodontic treatment or impaction. Exclusion criteria for all participants included systemic diseases affecting periodontal health, pregnancy or lactation, use of antibiotics, anti-inflammatory, or immunosuppressive drugs within the previous 3 months, and smoking or alcohol abuse. Inflamed gingival tissues from PD patients were obtained from the periodontal pocket area during flap surgery. All specimens were immediately preserved in RNAlater or fixed in 4% paraformaldehyde for subsequent analysis. The study was approved by the Ethics Committee of Anhui Medical University (Approval No. 2021006), and written informed consent was obtained from all participants.

### 2.14 Cell culture

Gingival tissues were harvested following standard procedures, washed with PBS, and minced into small fragments. These fragments were cultured in α-MEM (Gibco, USA) supplemented with 10% fetal bovine serum (OriCell, China) at 37°C with 5% CO_2_. Primary cells migrated from tissue explants after about seven days. Once 80% confluence was reached, cells were passaged and expanded. Cells between passages 4 and 8 were used for experiments. To mimic periodontal inflammation *in vitro*, HGFs were treated with *Porphyromonas gingivalis* lipopolysaccharide (*Pg*-LPS; InvivoGen, France) at a concentration of 1 μg/mL for 6 hours. This concentration was selected based on prior literature demonstrating effective induction of inflammatory responses without causing non-specific cytotoxicity (24, 25).

### 2.15 Quantitative real-time PCR

Total RNA was extracted using TRIzol reagent following the manufacturer’s protocol. cDNA was synthesized using a reverse transcription kit. Quantitative PCR was conducted with SYBR Green Master Mix (TaKaRa) on a QuantStudio 5 Real-Time PCR System. Gene expression levels were normalized to the internal control GAPDH. Primer sequences for the target genes and GAPDH are listed in **Supplementary Table 5**.

### 2.16 Mouse model of PD

Twelve wild-type (WT) C57BL/6 mice, purchased from Anhui Medical University, were randomly assigned to control and PD groups (n = 6 per group). Mice were anesthetized via intraperitoneal injection of sodium pentobarbital (40 mg/kg). In the PD group, PD was induced by carefully placing a sterile 5–0 silk ligature around the maxillary second molar under adequate illumination, without administration of *Pg*-LPS. The ligature was maintained for 10 days to allow disease development. All animal procedures were approved by the Ethics Committee of Anhui Medical University (Approval No. LLSC20250965) and conducted in accordance with institutional guidelines for the care and use of laboratory animals. Maxillary tissues were harvested and fixed in 4% paraformaldehyde for 24–48 hours, followed by decalcification and paraffin embedding for subsequent analyses.

### 2.17 Hematoxylin and eosin staining

H&E staining was performed on paraffin-embedded sections to assess histopathological changes. Stained slides were scanned using a panoramic digital slide scanner, and images were analyzed to evaluate inflammatory infiltration and alveolar bone loss.

### 2.18 Immunofluorescence staining

Cells were fixed with 4% paraformaldehyde for 20 minutes at room temperature, then washed with PBS. Tissue sections were first deparaffinized through a series of xylene and graded ethanol washes, followed by rehydration in PBS. Both cells and tissue sections were permeabilized with 0.1% Triton X-100 for 10 minutes and blocked with 5% BSA for 1 hour at room temperature. Samples were then incubated overnight at 4°C with primary antibodies. After washing with PBS, Alexa Fluor-conjugated secondary antibodies (1:100 dilution) were applied for 2 hours at room temperature in the dark. Nuclei were counterstained with DAPI (1 μg/mL) for 5 minutes. Fluorescence images were acquired using a Leica upright fluorescence microscope with consistent exposure settings. For quantification, at least three randomly selected fields per sample were analyzed using ImageJ software (version 1.48). The number of cells positive for both markers (co-stained cells) and the total number of nuclei (DAPI-stained) within each field were counted manually or by thresholding. The proportion of co-stained cells relative to total cells was calculated and averaged across fields for each sample.

### 2.19 Immunohistochemistry staining

Paraffin sections (4 μm) were deparaffinized, rehydrated, and antigen-retrieved in sodium citrate buffer at 95°C for 15 minutes. After blocking with 3% H_2_O_2_ and 5% goat serum, sections were incubated overnight with primary antibodies. Following secondary antibody incubation, staining was developed using a DAB kit and counterstained with hematoxylin. Images were captured and analyzed with ImageJ (v1.48).

### 2.20 Statistical analysis

Statistical analyses were performed using R software (version 4.3.1). Quantitative data from qPCR, immunofluorescence, and other assays are expressed as mean ± standard deviation (SD). Group comparisons were performed using unpaired two-tailed Student’s t-tests for comparisons between two groups, or one-way analysis of variance (ANOVA) followed by Tukey’s *post hoc* test for multiple group comparisons, where appropriate. For differential gene expression and pathway enrichment analyses, the BH procedure was applied to control the false discovery rate (FDR). Adjusted p-values less than 0.05 were considered statistically significant. For correlation analyses, Spearman’s rank correlation coefficient was calculated. All tests were two-sided unless otherwise specified. Data visualization was performed using the ggplot2 R package (version 3.5.1).

## 3 Results

### 3.1 scRNA-seq analysis revealed dysregulation of multiple PANoptosis and their related pathways in HGFs

**Figure 1:**
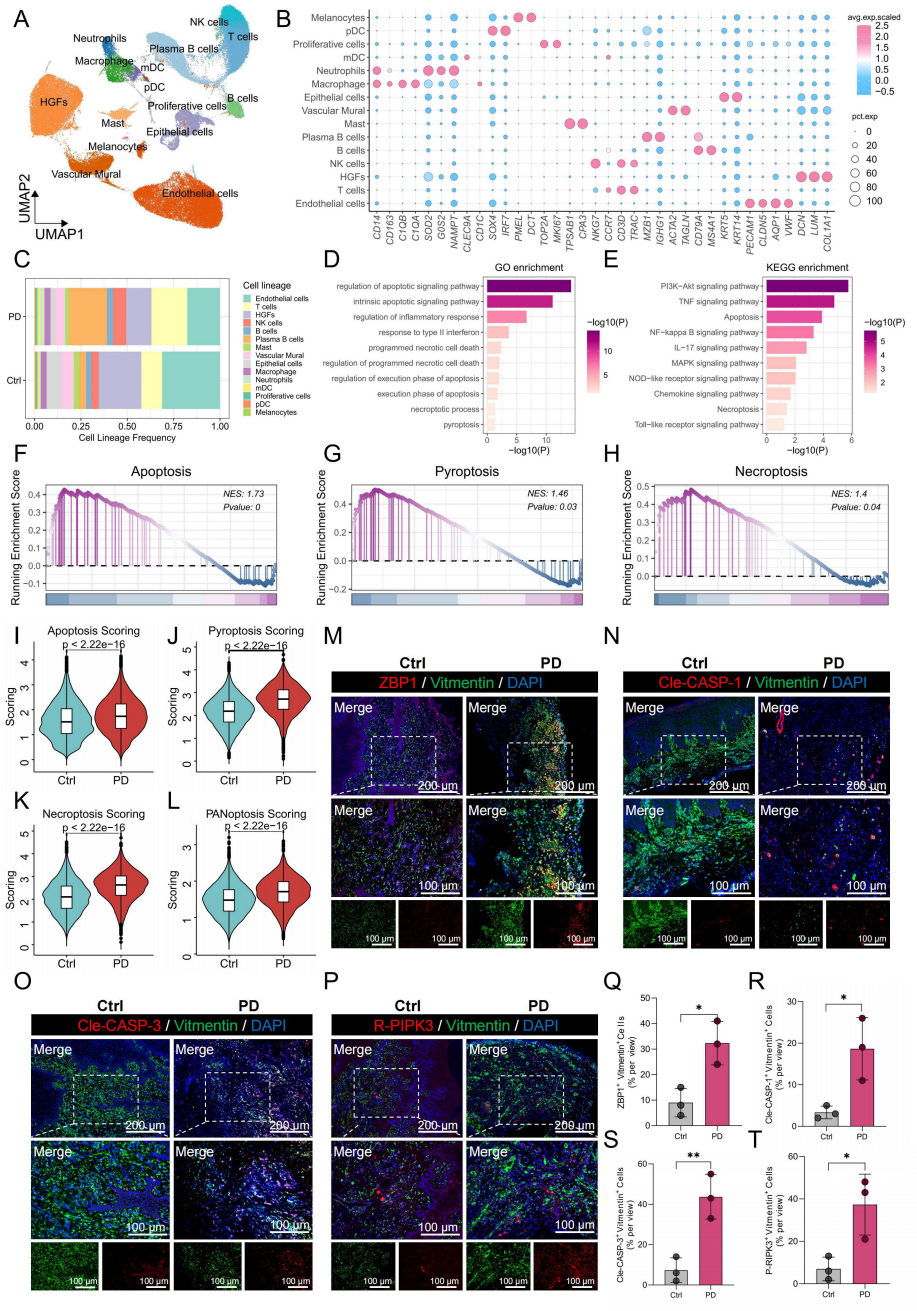
scRNA-seq revealed higher PANoptosis activity in PD HGFs. **(A)** UMAP plot showing the distribution of 15 distinct cell types in gingival tissues. **(B)** Bubble maps were used to display surface-annotated genes for various cell types. **(C)** Cell proportions of 15 cell types originating from normal and PD samples. **(D, E)** GO and KEGG enrichment analyses of DEGs in HGFs from healthy and PD samples. **(F-H)** GSEA was performed to assess the enrichment of apoptosis, pyroptosis, and necroptosis pathways in HGFs from healthy and PD samples. **(I-L)** Comparison of apoptotic, pyroptotic, necroptotic, and PANoptotic activities in HGFs from healthy and PD samples based on five different gene set scoring methods. **(M-P)** Immunofluorescence staining of ZBP1, cleaved CASP-1, cleaved CASP-3, and P-RIPK3, with vimentin-positive cells indicating HGFs. **(Q-T)** Quantification of fluorescence intensity for ZBP1, cleaved CASP-1, cleaved CASP-3, and RIPK3 in vimentin-positive HGFs from healthy and PD samples. HGFs: Human gingival fibroblasts; DEGs, differentially expressed genes; *p < 0.05, **p < 0.01. PD: Periodontitis.

To investigate the cellular composition of gingival tissues in PD, we performed single-cell RNA sequencing (scRNA-seq) on samples from both healthy controls and PD patients. After quality control and batch correction, 15 major cell populations were identified based on the expression of canonical lineage-specific markers (**Figures 1A, B**). These included HGFs (COL1A1, LUM, DCN), epithelial cells (KRT5, KRT14), endothelial cells (VWF, AQP1, CLDN5, PECAM1), vascular mural cells (TAGLN, ACTA2), melanocytes (DCT, PMEL), proliferating cells (MKI67, TOP2A), T cells (CCR7, CD3D, TRAC), B cells (MS4A1, CD79A), plasma B cells (MZB1, IGHG1), NK cells (NKG7, CD3D, TRAC), macrophages (CD14, CD163, C1QA), neutrophils (G0S2, SOD2, NAMPT), mast cells (CPA3, TPSAB1), myeloid dendritic cells (mDCs; CLEC9A), and plasmacytoid dendritic cells (pDCs; IRF7, SOX4). **Figure 1C** illustrates the relative abundances of these populations in healthy and PD samples. Notably, HGFs and endothelial cells were markedly reduced in PD, whereas immune cell subsets such as T cells, NK cells, plasma B cells, and neutrophils were relatively enriched, indicating a shift toward a pro-inflammatory microenvironment. Given the substantial decrease in HGF abundance and their potential contribution to PD pathogenesis, we further analyzed DEGs in HGFs between healthy and diseased tissues. The analysis revealed significant enrichment of cell death-related pathways including apoptosis, pyroptosis and necroptosis, suggesting that dysregulated cell death contributed to the progression of PD (**Figures 1D, E**). To validate these findings, we evaluated the activity of apoptosis, pyroptosis, and necroptosis in HGFs using GSEA and five independent gene set scoring algorithms. Consistently, HGFs from PD samples exhibited increased activation of these pathways compared to those from healthy controls (**Figures 1F-K**).

Given the convergence of apoptosis, pyroptosis, and necroptosis within the PANoptosis framework, we further investigated the potential involvement of PANoptosis in PD. PANoptosis activity was quantified using five independent gene set scoring methods based on the expression profiles of 109 PRGs. The analysis demonstrated a significant increase in PANoptotic activity in HGFs from PD samples compared to healthy controls (**Figure 1L**). To validate these results, immunofluorescence staining was performed on gingival tissues, assessing the co-localization of PANoptosis markers—ZBP1, cleaved-Caspase-1 (Cle-CASP-1), cleaved Caspase-3 (Cle-CASP-3), and P-RIPK3—with the HGFs marker vimentin. HGFs in PD samples showed markedly elevated expression of these markers relative to healthy gingiva, with quantitative fluorescence intensity analysis shown in **Figures 1M-P**. Quantitative analysis of fluorescence intensity confirmed these observations, with significantly higher levels of ZBP1, Cle-CASP-1, Cle-CASP-3, and P-RIPK3 in PD HGFs compared to healthy controls (**Figures 1Q-T**).

### 3.2 *In vitro* and *in vivo* validation confirmed PANoptosis activation in gingival fibroblasts

**Figure 2:**
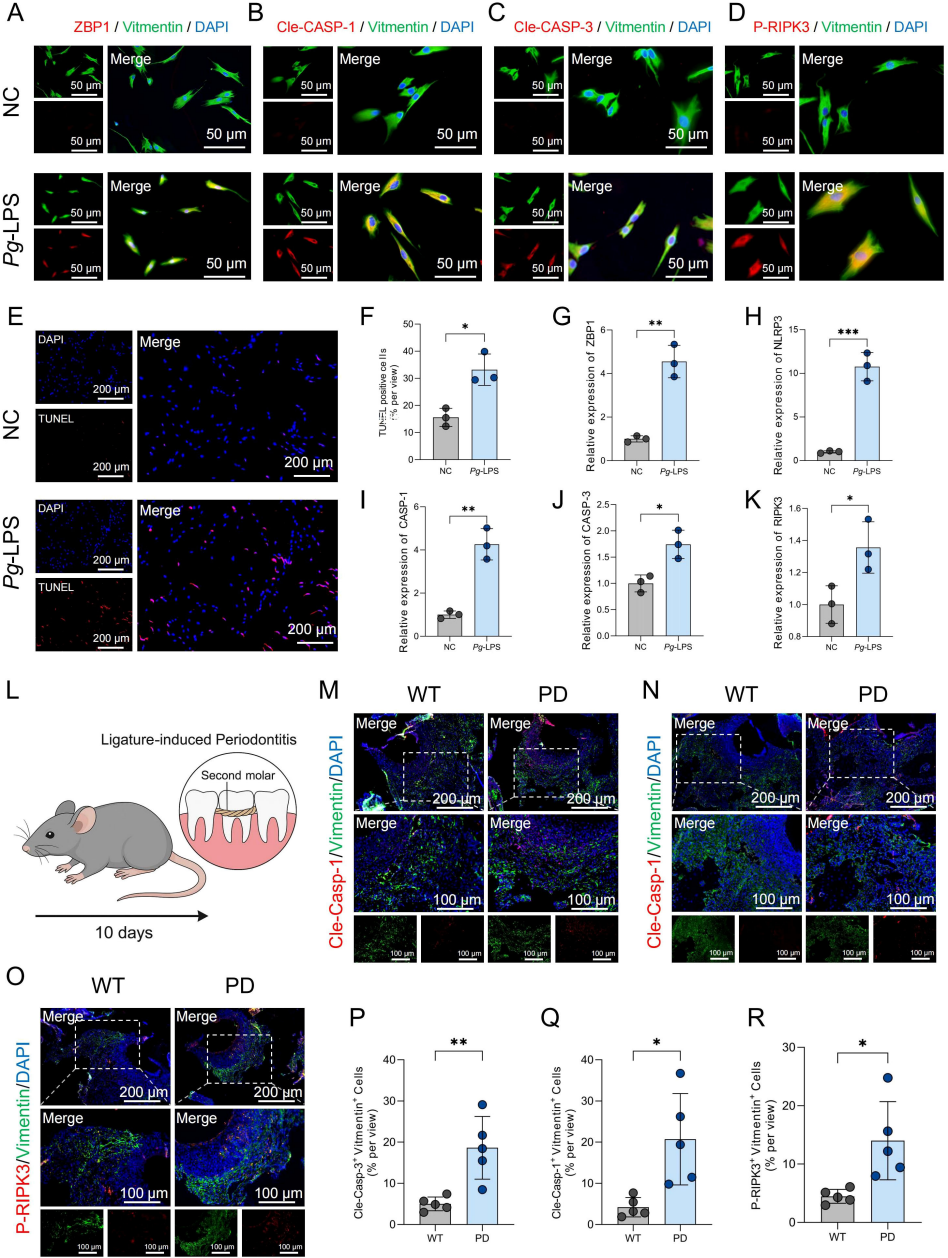
PANoptosis-related markers are upregulated in HGFs upon LPS stimulation and in gingival tissues of PD model mice. **(A-D)** Immunofluorescence staining showing the expression of ZBP1, cleaved CASP-1, cleaved CASP-3, and P-RIPK3 in HGFs following treatment with negative control (NC) or LPS. For each panel, the left image shows the individual channels prior to merging, and the right image shows the merged channels. **(E)** TUNEL staining of HGFs following stimulation with NC or LPS. **(F)** Quantification of TUNEL-positive HGFs following NC or LPS treatment. **(G-K)** qPCR analysis of mRNA expression levels of ZBP1, NLRP3, CASP-1, CASP-3 and RIPK3 in HGFs after NC or LPS treatment. **(L)** Schematic diagram illustrating the workflow for establishing the periodontitis mouse model. **(M-O)** Immunofluorescence results showing increased expression level of Cle-Casp-1, Cle-Casp-1, P-RIPK3 in gingival fibroblasts from periodontitis mouse gingival tissues. **(P-R)** Quantification of fluorescence intensity for Cle-Casp-1, Cle-Casp-3, and P-RIPK3 in gingival fibroblasts. *p < 0.05, **p < 0.01, ***p < 0.001. HGFs: human gingival fibroblasts.

Additionally, primary HGFs were cultured *in vitro* and stimulated with lipopolysaccharide (LPS). Immunofluorescence staining revealed increased expression of the same PANoptosis-related markers in LPS-treated HGFs compared to unstimulated controls (**Figures 2A-D**). Furthermore, TUNEL staining demonstrated a significant increase in DNA fragmentation in LPS-treated HGFs, indicative of cell death (**Figure 2E**), and quantitative analysis confirmed a higher percentage of TUNEL-positive cells following LPS stimulation (**Figure 2F**). Consistent with these findings, qPCR analysis showed upregulation of key PANoptosis-related genes—including ZBP1, NLRP3, CASP-1, CASP-3 and RIPK3—following LPS treatment (**Figures 2G-K**). These findings collectively indicate that PANoptosis activation in HGFs may contribute to the development of PD.

To further validate the involvement of PANoptosis in the progression of PD, an *in vivo* PD model was established in mice by ligating the maxillary second molars for 10 days (**Figure 2L**). H&E staining confirmed successful induction of PD, as evidenced by inflammatory infiltration and alveolar bone loss (**Supplementary Figure 1**). Immunofluorescence staining was then performed to assess the expression of PANoptosis-related markers in gingival fibroblasts. Compared to wild-type controls, gingival fibroblasts from PD mice exhibited increased expression of Cle-Casp-1, Cle-Casp-3, and P-RIPK3 (**Figures 2M-O**). Quantitative analysis of immunofluorescence intensity further confirmed significantly elevated levels of these markers in the PD group (**Figures 2P-R**), suggesting the activation of PANoptosis in the gingival microenvironment during PD.

### 3.3 PANoptosis participates in immune regulation

**Figure 3:**
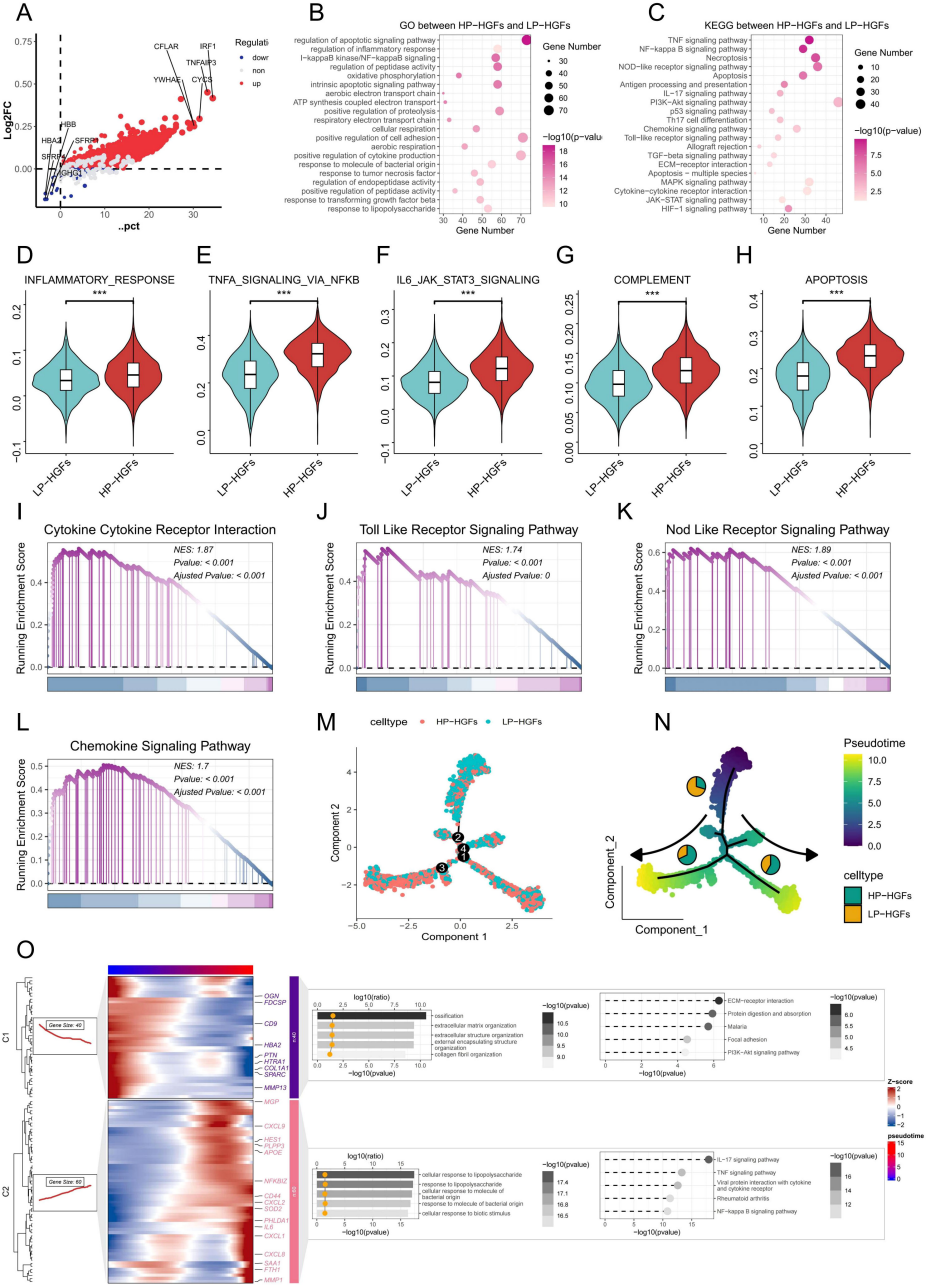
Molecular characteristics and pathway alterations between HP-HGFs and LP-HGFs. **(A)** DEGs between HP-HGFs and LP-HGFs. **(B, C)** GO and KEGG enrichment analyses of DEGs between HP-HGFs and LP-HGFs. **(D-H)** Comparison of hallmark gene set activity between HP-HGFs and LP-HGFs using GSVA. **(I-L)** GSEA analysis of the cp:KEGG pathway gene sets to assess signaling differences between the two HGF subsets. **(M)** Cell type distribution of HP-HGFs and LP-HGFs along pseudotime trajectory branches. **(N)** A pseudotime trajectory was constructed to investigate the dynamic transcriptional states of HGFs categorized by PANoptosis activity. Cells were colored based on pseudotime values, ranging from early (purple) to late (yellow) developmental stages, as indicated by the color bar. The trajectory demonstrates a branched topology, suggestive of distinct differentiation or activation paths. Cells were further annotated by PANoptosis score-based classification into HP-HGFs (cyan) and LP-HGFs (orange), and the proportion of each cell type within the major trajectory branches is represented by the embedded pie charts. Arrows indicate the direction of pseudotime progression along each branch. **(O)** Branched heatmap showing dynamic gene expression and functional enrichment along pseudotime trajectory in HGFs associated with PANoptosis. Heatmap shows two major gene modules (C1 and C2) identified along the pseudotime trajectory of HGFs using Monocle2. Each row represents a gene and each column a cell ordered by pseudotime, with color indicating Z-score-scaled expression levels. Cells are aligned according to pseudotime (top color bar), and genes are grouped by shared dynamic expression patterns. Functional enrichment analysis (GO and KEGG) is visualized using dot plots overlaid on the heatmap. The color gradient and bar length represent pathway significance (-log10 p-value), while the x-coordinate of the yellow dots indicates the enrichment magnitude (log10 ratio), collectively highlighting distinct biological processes and signaling pathways associated with each gene module and reflecting transcriptional heterogeneity during PANoptosis-related transitions. *p < 0.05, **p < 0.01, ***p < 0.001. HGFs: human gingival fibroblasts; DEG: Differentially expressed genes; HP-HGF: high-PANoptosis HGFs; LP-HGFs: low-PANoptosis HGFs.

To further investigate the functional characteristics and signaling pathways associated with PANoptosis activity, we stratified HGFs into high-PANoptosis (HP) and low-PANoptosis (LP) subgroups based on their PANoptosis scores. As shown in **Figure 3A**, differential gene expression analysis revealed distinct gene profiles between HP and LP subgroups of HGFs. Functional enrichment analysis showed that the differentially expressed genes (DEGs) were significantly associated with inflammation and immune-related pathways, such as the inflammatory response, NF-κB signaling, cytokine–cytokine receptor interaction, and chemokine signaling (**Figures 3B, C**). Moreover, hallmark pathways—including TNFA signaling via NF-κB, IL6-JAK-STAT3 signaling, complement activation, apoptosis, and interferon-γ response—were markedly enriched in HP-HGFs (**Figures 3D-H**). GSEA further confirmed significant upregulation of pathways like Toll-like receptor signaling, NOD-like receptor signaling, and chemokine signaling in HP-HGFs compared to LP-HGFs (**Figures 3I-L**). Subsequently, we employed pseudotime trajectory analysis to investigate the dynamic changes of HGFs with high and low PANoptosis activity during cellular development. The results revealed that LP-HGFs were primarily located at early developmental stages, while HP-HGFs were enriched in later phases, suggesting a temporal progression toward a high PANoptotic state (**Figure 3M**). Correspondingly, as HGFs transitioned along the pseudotime trajectory, the proportion of LP-HGFs gradually declined, whereas HP-HGFs became increasingly dominant (**Figure 3N**). This phenotypic shift was accompanied by a gradual increase in the expression of pro-inflammatory cytokines and chemokines, as well as the activation of immune-related pathways, including responses to bacterial products and lipopolysaccharide, TNF signaling, NF-κB, and IL-17 pathways (**Figure 3O**). Collectively, these findings indicate that PANoptosis activation in HGFs is closely linked to pro-inflammatory signaling and may contribute to the immune dysregulation and tissue destruction characteristic of PD.

### 3.4 Enhanced cell-cell interactions in HP-HGFs

**Figure 4:**
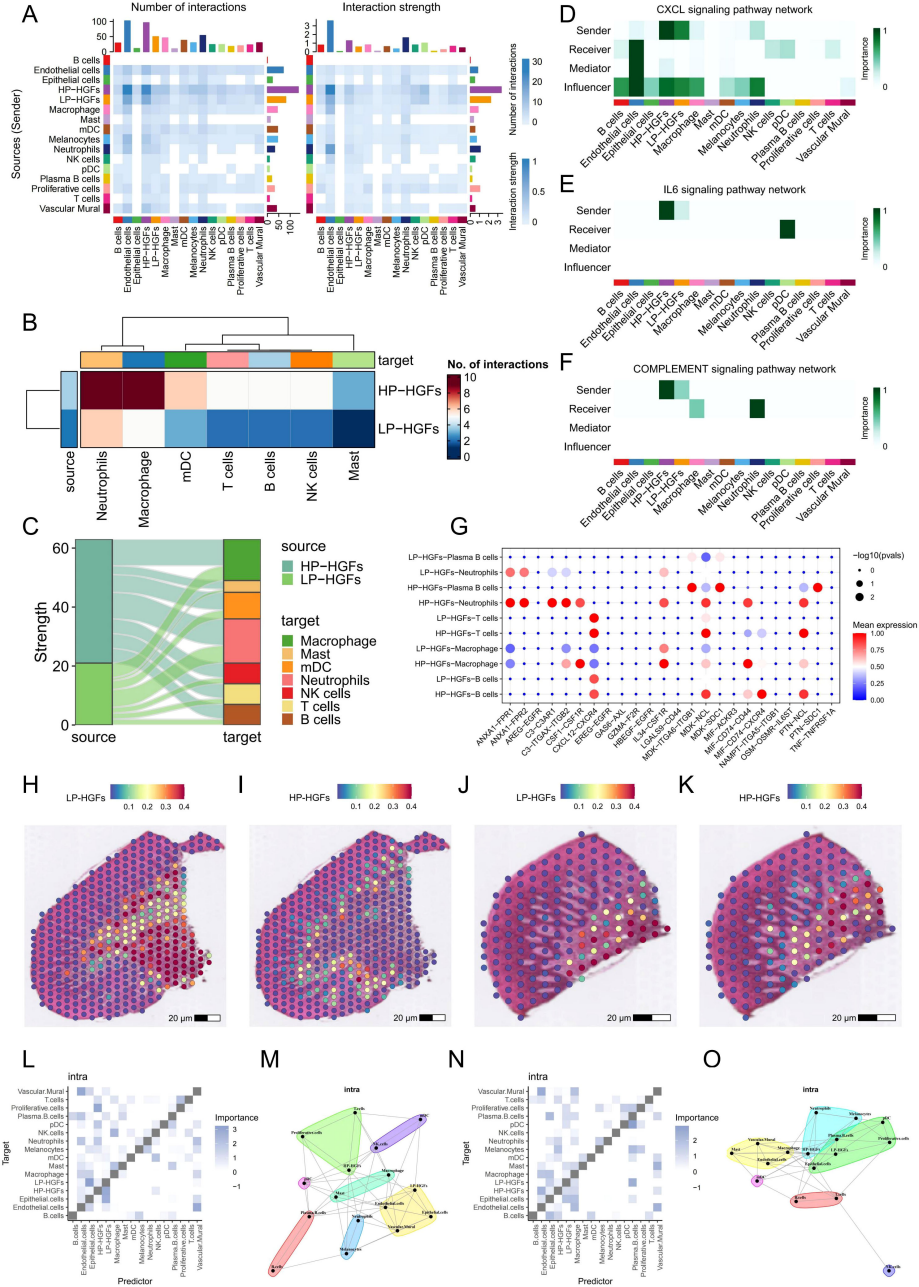
Cell-cell communication analysis between HP-HGFs and LP-HGFs. **(A)** Heatmap illustrating the strength and number of interactions between HP-HGFs and LP-HGFs. **(B, C)** Heatmap and mulberry plot depicting interaction intensity between HP-HGFs or LP-HGFs as sender cells and various immune cell populations. **(D-F)** Heatmaps showing the relative contribution of each cell type within the CXCL, IL6 and complement signaling networks. **(G)** Bubble plot displaying significant ligand-receptor pairs for HP-HGFs and LP-HGFs, respectively. **(H, I)** Spatial transcriptomic maps showing the localization of HP-HGFs and LP-HGFs in sample GSM6258258. **(J, K)** Spatial transcriptomic maps showing the localization of HP-HGFs and LP-HGFs in sample GSM6258257. **(L, M)** Spatial analysis of long-range intercellular interactions among various cell types in GSM6258258. **(N, O)** Spatial analysis of long-range intercellular interactions among various cell types in GSM6258257. HP-HGF: high-PANoptosis HGFs; LP-HGFs: low-PANoptosis HGFs.

To investigate changes in intercellular communication associated with PANoptosis activity, we performed CellChat analysis comparing HP-HGFs and LP-HGFs. The results revealed that HP-HGFs exhibited markedly enhanced communication with various immune cell types—including neutrophils, T cells, B cells, macrophages, mast cells, and plasma cells—suggesting a heightened immunomodulatory role (**Figures 4A-C**). Notably, HP-HGFs demonstrated stronger outgoing signaling through key pathways such as CXCL, IL6, and the complement system, indicating active involvement in immune cell recruitment and activation (**Figures 4D-F**). Compared to LP-HGFs, HP-HGFs exhibited markedly increased ligand–receptor interactions, such as CSF1–CSF1R, IL34–CSF1R, and PTN–NCL, particularly targeting B cells, T cells, neutrophils, and macrophages (**Figures 4G**). These results suggest that HGFs with active PANoptosis significantly influence the inflammatory milieu in PD by enhancing immune cell recruitment and intensifying pro-inflammatory signaling, potentially contributing to sustained inflammation and disease progression.

### 3.5 ST data highlights stronger immune interaction of HP-HGFs

To further elucidate the role of PANoptosis in PD, we integrated spatial transcriptomics data from two PD samples, GSM6258256 and GSM6258257. After filtering low-quality data, we applied the “SCTransform” method for normalization and batch correction, followed by dimensionality reduction. The spatial feature visualization (**Supplementary Figures 2A, B**) illustrated the distribution of the number of genes detected at each capture spot (nFeature_Spatial) across the tissue sections. Subsequent clustering analysis identified six and five distinct spatial domains in the two samples (**Supplementary Figures 2C, D**), respectively, highlighting the potential spatial heterogeneity of PD lesions. We then performed deconvolution using scRNA-seq results and projected the inferred cell types onto the spatial transcriptomics framework. Both samples showed highly consistent results, revealing significant spatial heterogeneity in the localization of HP and LP subpopulations of HGFs (**Figures 4H-K**). Spatial analysis of cell-cell interactions through co-localization revealed that, in PD tissues, HP-HGFs tended to form long-distance interactions with immune cells including neutrophils, T cells, and B cells (**Figures 4L-O**). This suggests a significant role for HP-HGFs in regulating the local immune microenvironment. These spatial transcriptomics data align with our scRNA-seq findings, reinforcing the idea that PANoptosis may contribute to PD development by modulating immune activity within the tissue niche.

### 3.6 Bulk RNA-seq reveals elevated PANoptosis levels and identification of differentially expressed PANoptosis-related genes in PD patients

**Figure 5:**
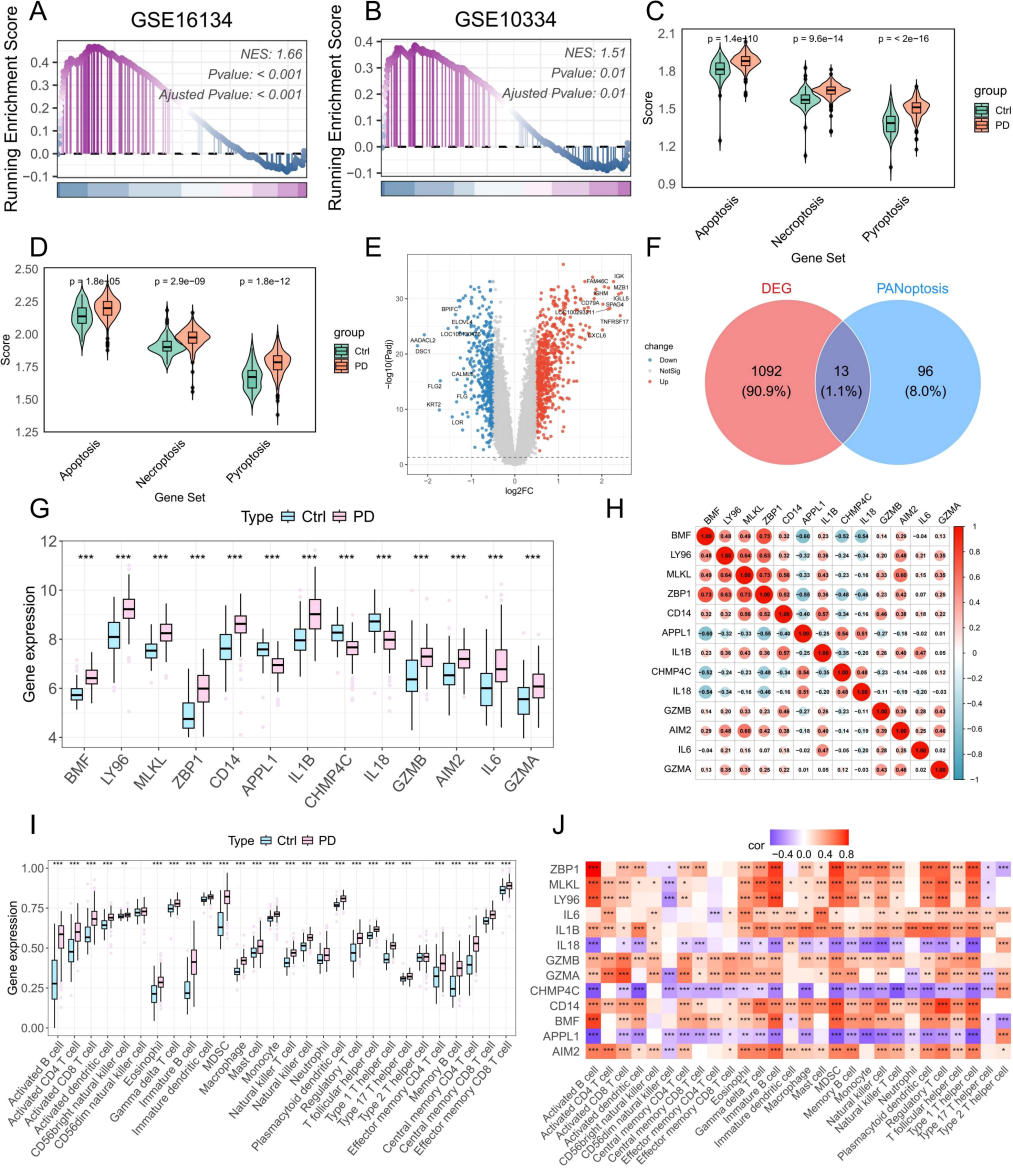
Bulk RNA-seq analysis reveals increased PANoptotic activity in PD samples and identifies DE-PRGs. **(A, B)** GSEA showing increased PANoptotic activity in PD samples compared to healthy controls in datasets GSE16134 and GSE10334. **(C, D)** GSVA assessing the activity of apoptotic, pyroptotic, and necroptotic pathways in healthy and PD samples from GSE16134 and GSE10334. **(E)** Volcano plot illustrating DEGs between healthy and PD samples in GSE16134. **(F)** Venn diagram showing the intersection of DEGs and PRGs. **(G)** Boxplots showing the expression levels of DE-PRGs. **(H)** Correlation analysis of 13 DE-PRGs. **(I)** ssGSEA-based estimation of the infiltration levels of 28 immune cell types in healthy and PD samples. **(J)** Correlation analysis between DE-PRGs and the infiltration abundance of 28 immune cell types. DEGs: differentially expressed genes; DE-PRGs: differentially expressed PANoptosis-related genes; *p < 0.05, **p < 0.01, ***p < 0.001. PD: Periodontitis.

Meanwhile, we investigated the role of PANoptosis in PD at the bulk RNA-seq level. GSEA of two independent PD datasets revealed that PANoptotic activity was significantly elevated in PD samples compared to healthy controls (**Figures 5A, B**). Furthermore, three key cell death-related pathways—apoptosis, pyroptosis, and necroptosis—were also markedly dysregulated between healthy and PD groups (**Figures 5C, D**). To identify PANoptosis-related DEGs, we first screened 1,106 DEGs between healthy and PD samples (**Figure 5E**). Cross-referencing these with a curated list of PANoptosis-associated genes yielded 13 overlapping candidates (**Figure 5F**). Their expression patterns across groups are presented in **Figure 5G**, and strong correlations among these genes are depicted in **Figure 5H**. To explore immune alterations, we assessed immune cell infiltration using ssGSEA and observed a marked increase in multiple immune cell populations in PD samples compared to healthy controls (**Figure 5I**). Correlation studies demonstrated significant associations between several PANoptosis genes and immune cell infiltration (**Figure 5J**). Collectively, these results indicate that PRGs are closely intertwined with immune activation, potentially driving the pro-inflammatory environment characteristic of PD.

### 3.7 Consensus clustering and immune characteristics among subgroups

**Figure 6:**
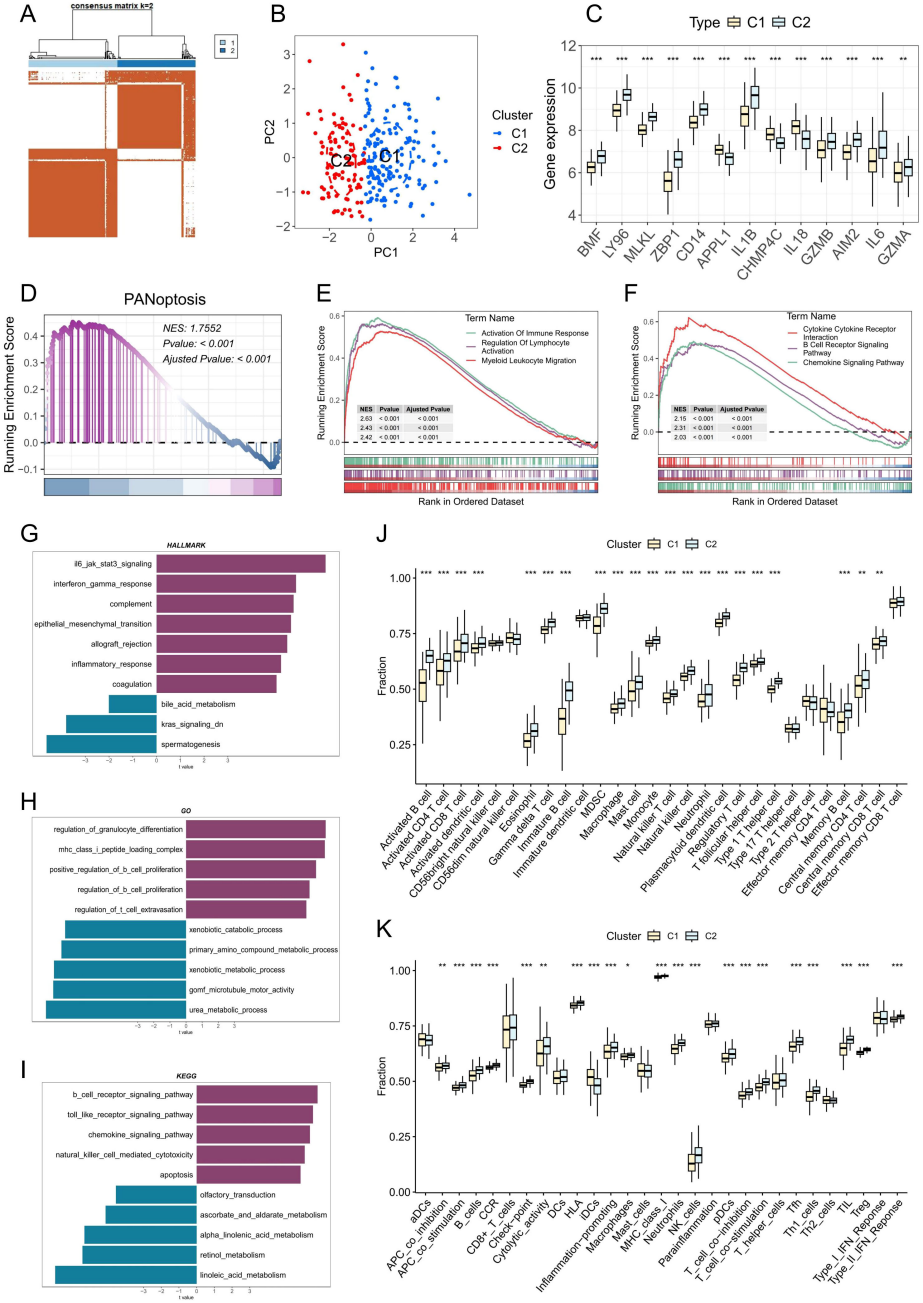
Identification and characterization of PANoptosis-related molecular subtypes in PD. **(A)** Consensus clustering heatmap when k= 2, indicating the optimal classification into two PANoptosis-related clusters. **(B)** Principal component analysis (PCA) showing clear separation between the two PANoptosis clusters. **(C)** Boxplots displaying the expression levels of DE-PRGs between the two clusters. **(D)** GSEA showing differences in PANoptotic activity between the two clusters. **(E, F)** GSEA identifying significant differences in GO terms and KEGG pathways between the two clusters. **(G-I)** GSVA illustrating variations in hallmark, GO, and KEGG gene sets between the two clusters. **(J)** Estimated proportion of immune cell infiltration in the two clusters. **(K)** Differences in immune-related functional scores between the two clusters; *p < 0.05, **p < 0.01, ***p < 0.001. PD: Periodontitis.

To investigate the immunological characteristics and functional implications of PANoptosis in PD, we stratified patient samples based on the expression patterns of PRGs using consensus clustering. The optimal number of clusters was determined through evaluation of the cumulative distribution function (CDF) curves (**Supplementary Figure 3**) and consensus matrix heatmaps, ultimately identifying two distinct molecular subtypes (**Figure 6A**). Analysis of PCA further validated the robustness and reliability of this classification (**Figure 6B**). As shown in **Figure 6C**, multiple PANoptosis-associated genes were significantly upregulated in the C2 subtype, suggesting enhanced PANoptotic signaling in this group. To quantify PANoptotic activity, GSEA was performed between the two subtypes. The results demonstrated that the C2 subtype exhibited markedly elevated PANoptosis activity compared to the C1 subtype (**Figure 6D**). To elucidate the biological functions and pathways associated with PANoptosis, we performed GSEA on two distinct PANoptosis-related subtypes. The C2 subtype exhibited significant upregulation of multiple immune-related pathways, including immune response activation, myeloid leukocyte migration, B cell receptor signaling, and chemokine signaling (**Figures 6E, F**). We also performed pathway enrichment analysis using gene sets from the Molecular Signatures Database (MSigDB), including the HALLMARK, C5-GO, and C2-KEGG collections. The C2 subtype demonstrated upregulation of multiple immuno-inflammatory pathways, such as IL6–JAK–STAT3 signaling, inflammatory response, and complement in the HALLMARK set; regulation of myeloid cell differentiation, regulation of B cell proliferation, and T cell extravasation in the GO set; and B cell receptor signaling, Toll-like receptor signaling, and chemokine signaling in the KEGG set (**Figures 6G–I**). Collectively, these results suggest a strong association between PANoptosis and immune activation. Further analysis of immune cell infiltration and immune-related functions revealed a broadly elevated immune status in the C2 subtype, characterized by increased infiltration of various immune cells and enhanced immune functional activities (**Figures 6J, K**). Taken together, these findings underscore the pivotal role of PANoptosis-related molecular features in modulating the immune microenvironment of PD and provide novel insights into the inflammatory pathogenesis of the disease.

### 3.8 WGCNA network construction and identification of key modules

**Figure 7:**
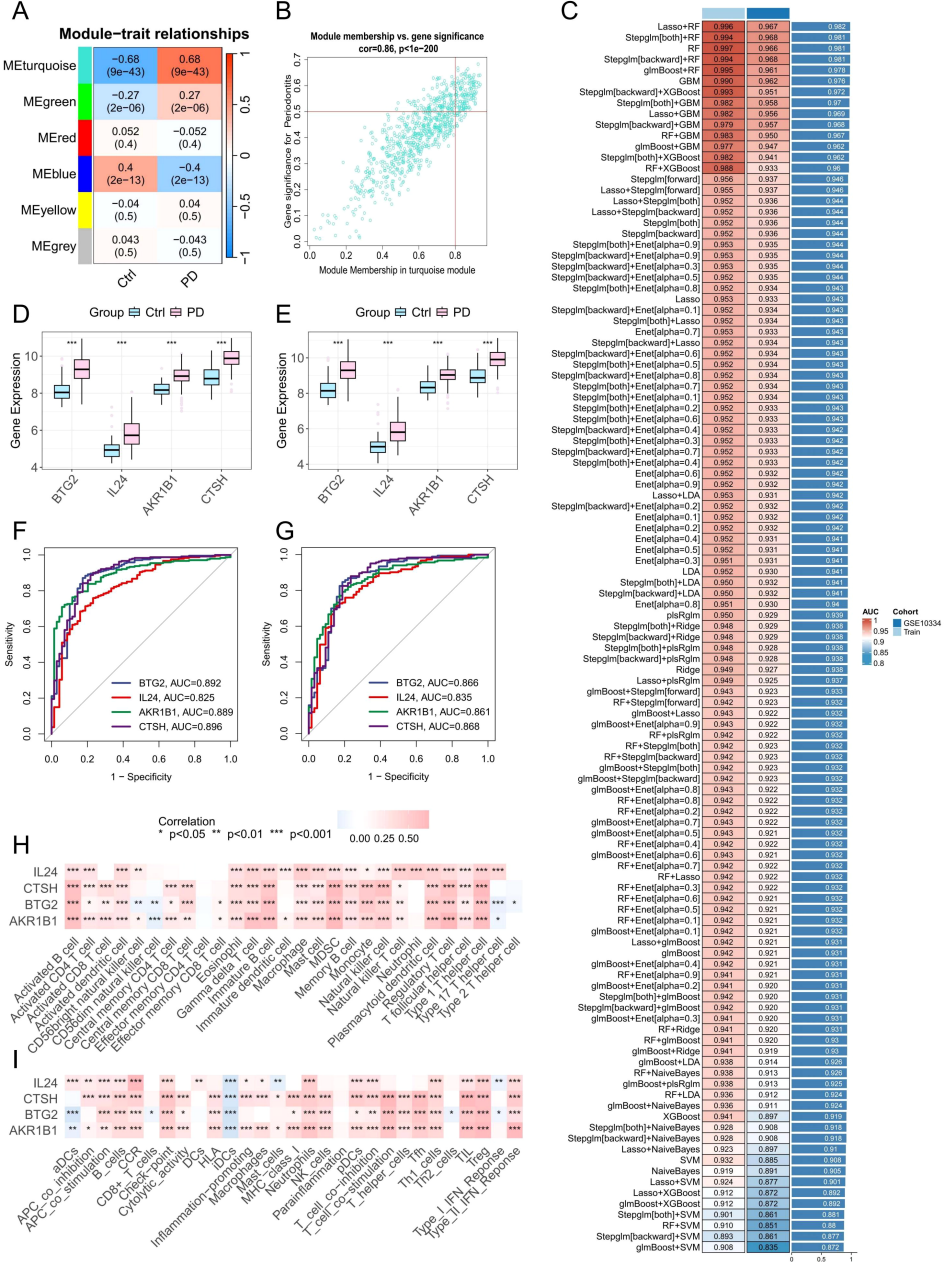
Identification of potential PANoptosis-related hub genes and model construction. **(A)** Heatmap showing module-trait correlations generated by WGCNA analysis. **(B)** Scatter plot depicting the relationship between gene significance for PD and module membership in the MEturquoise module. **(C)** Performance of 113 machine learning algorithm combinations evaluated using 10-fold cross-validation. **(D, E)** Boxplots showing the expression levels of identified core PANoptosis-related genes in datasets GSE16134 and GSE10334. **(F, G)** ROC curves assessing the diagnostic performance of the core genes in the training set (GSE16134) and validation set (GSE10334). **(H, I)** Correlation analysis between the four core PANoptosis-related genes and immune cell infiltration as well as immune-related functional pathways.

To investigate gene co-expression patterns associated with PD, we applied Weighted Gene Co-Expression Network Analysis (WGCNA). Following hierarchical clustering to exclude outlier samples, a soft-threshold power of 5 (β = 5) was chosen to achieve a scale-free network structure. This analysis identified 6 distinct gene modules, among which the turquoise module exhibited the highest positive correlation with disease status (r = 0.68), encompassing 690 genes (**Figure 7A**). Subsequent evaluation of gene significance versus module membership demonstrated a strong association, underscoring the importance of these genes for further functional analyses (**Figure 7B**).

### 3.9 Screening of characteristic genes related to PANoptosis and construction of machine learning

At the bulk RNA-seq level, 343 upregulated and 115 downregulated DEGs related to PANoptosis were identified between the two subtypes (**Supplementary Table 6**). Meanwhile, scRNA-seq analysis revealed 245 upregulated and 5 downregulated DEGs distinguishing HP-HGFs from LP-HGFs (**Supplementary Table 7**). By intersecting these gene sets with results from WGCNA and DEGs identified between healthy and PD samples, we ultimately obtained 13 core PANoptosis-related genes (**Supplementary Figures 4A, B**). These genes were subsequently used to construct 113 machine learning models. The GSE16134 dataset served as the training set, while GSE10334 was used for external validation. The final model, which integrated LASSO and RF, demonstrated excellent predictive performance, achieving an AUC of 0.996 in the training cohort and 0.967 in the external validation cohort (**Figure 7C**). To further assess its diagnostic efficacy, additional metrics were calculated. In the training dataset (GSE16134), the model yielded a sensitivity of 98.8%, specificity of 89.9%, and an F1 score of 97.9%. In the independent validation dataset (GSE10334), it maintained a high sensitivity of 97.3% and an F1 score of 93.9%, with a moderate specificity of 81.9% (**Supplementary Figure 5**). These results highlight the robustness and clinical potential of the predictive model. LASSO regression identified 11 candidate genes. Subsequently, RF analysis refined the selection to 4 key genes (BTG2, CTSH, AKR1B1, and IL24), which contributed most significantly to the model’s predictive performance and stability.

### 3.10 Expression levels and diagnostic significance of hub genes

To assess the relevance of these hub genes in PD, we analyzed their expression levels and diagnostic performance. Boxplot analyses of the GSE16134 and GSE10334 datasets showed that the four hub genes were significantly upregulated in PD samples (**Figures 7D, E**). ROC curve analysis further demonstrated that all four genes exhibited strong diagnostic performance, with AUC values exceeding 0.8 in both datasets (**Figures 7F, G**), indicating their robust predictive value for PD.

### 3.11 Correlation between hub genes and immune infiltration

Pearson correlation analysis was conducted to explore the association between the expression of target genes and immune cell infiltration as well as immune-related functions. The findings showed positive correlations with the majority of immune cell types and immune activities (**Figures 7H, I**), indicating these genes may play important roles in modulating immune responses and influencing PD progression.

### 3.12 Functional enrichment analysis of key genes

To further clarify the biological functions of the four key genes (BTG2, CTSH, AKR1B1, and IL24) in PD, we conducted single-gene GSEA and GSVA analyses. GSVA indicated that these genes were positively associated with multiple immune and inflammatory pathways, including IL2/STAT5 and IL6/JAK/STAT3 signaling, interferon responses, complement activation, and inflammatory responses (**Supplementary Figures 6A-D**). GSEA further revealed that BTG2, CTSH, and AKR1B1 were enriched in B cell receptor and chemokine signaling pathways (**Supplementary Figures 6E-G**), while IL24 was primarily associated with cytokine–cytokine receptor interaction and the JAK-STAT signaling pathway (**Supplementary Figure 6H**). These findings imply that elevated expression of BTG2, CTSH, AKR1B1, and IL24 may play a role in driving immune dysregulation in PD.

### 3.13 Experimental validation of PANoptosis-related hub genes

**Figure 8:**
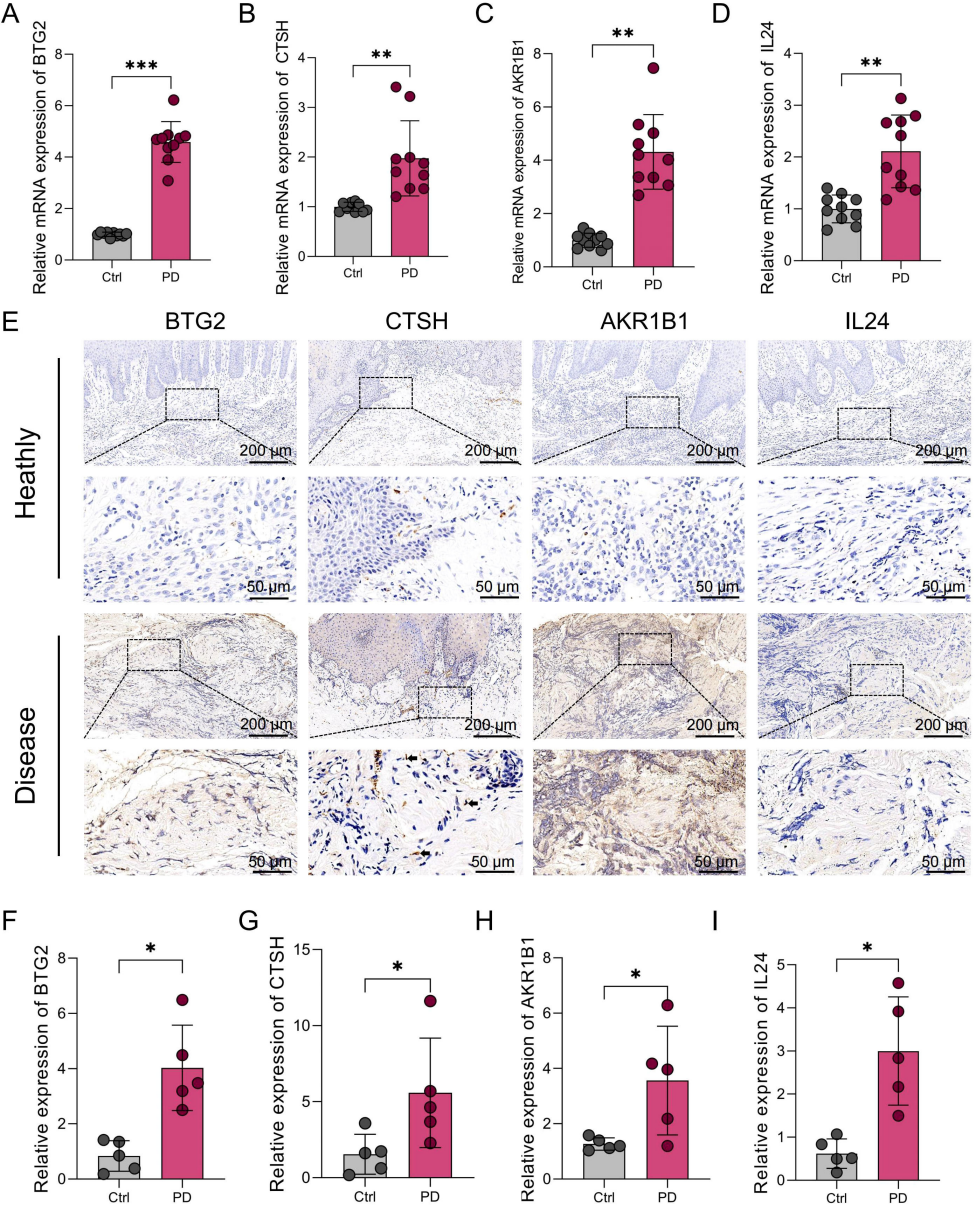
Expression levels of BTG2, CTSH, AKR1B1, and IL24 in gingival tissues from healthy individuals and PD patients. **(A-D)** qRT-PCR analysis showing the mRNA expression levels of BTG2, CTSH, AKR1B1, and IL24 in gingival tissues from human healthy controls (n=10) and PD patients (n=10). GAPDH was used as the internal control. **(E)** Immunohistochemical staining of BTG2, CTSH, AKR1B1, and IL24 in gingival tissues from human healthy controls and PD patients. Arrows indicate spindle-shaped HGFs. **(F-I)** Quantification of immunohistochemical staining intensity for each target gene. *p < 0.05, **p < 0.01, ***p < 0.001. PD: Periodontitis.

To validate our bioinformatics findings, four PANoptosis-associated hub genes (BTG2, CTSH, AKR1B1, and IL24) were selected for experimental confirmation. qPCR analysis revealed significant upregulation of these genes in PD gingival tissues compared to healthy controls (**Figures 8A-D**). Immunohistochemistry further confirmed consistent protein-level expression patterns (**Figure 8E**), and quantitative analysis demonstrated significantly increased staining intensity in PD samples (**Figures 8F-I**), supporting the robustness of the PANoptosis-based model. Notably, we found that CTSH was mainly localized in the epithelial layer and vascular-associated regions, suggesting that it may be involved in the occurrence of PANoptosis in these compartments. However, as the present study primarily focused on HGFs, these regions were not examined in detail, representing a limitation that warrants further investigation in future studies.

## 4 Discussion

PD is a chronic inflammatory condition marked by the gradual destruction of tooth-supporting tissues, primarily driven by dysregulated host immune responses to microbial biofilms (26). While traditional forms of PCD, such as apoptosis and necroptosis, have been implicated in periodontal tissue damage (4), the role of PANoptosis—a recently identified, highly inflammatory form of cell death that integrates pyroptosis, apoptosis, and necroptosis—remains largely unexplored in the context of periodontal disease (14). Given its unique capacity to orchestrate immune-inflammatory cascades, PANoptosis may represent a crucial mechanism linking cellular stress responses to immune activation in the periodontal microenvironment.

Our single-cell analysis revealed that HGFs in PD tissues exhibit transcriptional signatures indicative of multiple programmed cell death modalities, with PANoptosis-related genes significantly upregulated compared to healthy controls. Increasing evidence suggests that HGFs actively participate in inflammatory signaling during PD, beyond their traditional role as structural matrix components maintaining tissue integrity. Under inflammatory conditions, HGFs are susceptible to diverse forms of programmed cell death, which contribute to tissue destruction and disease progression. Apoptosis of HGFs, induced by pro-inflammatory cytokines such as TNF-α and IL-1β, has been well documented as a mechanism driving connective tissue degradation (27). Furthermore, recent studies demonstrate that microbial components can trigger pyroptosis in HGFs via caspase-1 activation, thereby amplifying local inflammatory responses (28). Emerging data also indicate that necroptosis may occur in HGFs under sustained inflammatory stress, further exacerbating tissue damage (20). Collectively, these findings highlight that HGFs in PD concurrently exhibit apoptotic, pyroptotic, and necroptotic death modalities. Our results extend this understanding by suggesting that these pathways may not act independently but rather operate in an integrated fashion characteristic of PANoptosis. This is consistent with emerging research in other inflammatory contexts, such as sepsis and viral infections, where PANoptosis orchestrates potent inflammatory responses through the simultaneous activation of pyroptotic, apoptotic, and necroptotic machinery (29, 30). Therefore, our study provides the first transcriptomic evidence identifying HGFs in PD as a previously underrecognized cellular source of PANoptotic activity.

Functionally, the spatial transcriptomic and cell–cell communication analyses revealed that HP-HGFs exhibited enhanced interactions with multiple immune cell populations, including neutrophils, T cells, and B cells. This observation suggests that PANoptotic HGFs may contribute to shaping the local immune landscape. The link between PANoptosis in HGFs and the amplified immune response can be understood through several mechanisms. PANoptosis leads to pore formation and plasma membrane rupture, resulting in the release of DAMPs and mature inflammatory cytokines such as IL-1β and IL-18 (30). These mediators are potent activators of innate immunity, recruiting and activating neutrophils and macrophages. Although direct evidence in HGFs is limited, similar immunogenic effects of PANoptosis have been observed in other contexts—including cancer and infectious models—where PANoptotic cell death enhances immune cell infiltration and inflammation (29, 31). Secondly, dying HGFs and their secretory factors may promote adaptive immune activation by releasing chemokines and pro-inflammatory cytokines that recruit and modulate T and B cells, which is consistent with our spatial transcriptomic evidence of enhanced interactions between HP-HGFs and these lymphocyte populations. Third, PANoptosis-associated release of inflammatory mediators and subsequent immune cell activation may enhance proteolytic activity within periodontal lesions—such as increased production of MMPs and other proteases—thereby linking molecular cell death events to extracellular matrix degradation and clinical tissue loss observed in PD (32). Overall, these observations suggest a plausible feed-forward loop: inflammatory stimulation induces PANoptosis in HGFs; PANoptotic cells amplify immune activation and proteolytic processes; and the resulting inflammation further stresses stromal cells, sustaining tissue damage. Importantly, these interpretations remain correlative and hypothesis-generating. To establish causality and therapeutic potential, targeted functional studies are needed—such as inhibiting key PANoptosome components (e.g., ZBP1 or RIPK3) or inflammasome signaling in HGFs, assessing consequent changes in immune recruitment and matrix destruction in co-culture and animal models, and validating key mediators at the protein level with multiplexed imaging.

Using an integrative machine learning pipeline combining 113 feature selection–classifier pairs, we first applied a primary algorithm (e.g., LASSO) to identify candidate variables, followed by a secondary algorithm (e.g., Random Forest) to construct classification models. Model performance was evaluated in both training and validation cohorts, and the combination with the highest mean AUC was selected as optimal. In this top-performing model, four PANoptosis-related hub genes—BTG2, CTSH, AKR1B1, and IL24—were consistently retained across algorithms and ranked highest in discriminative power, substantially improving model performance (AUC = 0.996 training; 0.967 validation).

Functionally, these genes may act as regulatory nodes linking inflammatory stress to programmed cell death pathways in the periodontal microenvironment. BTG2 is an anti-proliferative gene induced by oxidative and genotoxic stress, functioning as a downstream effector of p53 (33). It regulates the cell cycle by inducing G1/S and G2/M arrest and promotes apoptosis via upregulation of pro-apoptotic proteins such as Bax (34). BTG2 also participates in DNA damage repair and oxidative stress responses through both p53-dependent and ROS-NF-κB pathways (35, 36). Its expression may reflect cellular stress and apoptosis initiation under chronic inflammatory conditions, suggesting a role in the apoptotic component of PANoptosis in PD. CTSH encodes a lysosomal cysteine protease primarily involved in intracellular protein degradation and antigen processing, playing a vital role in lysosomal function and cellular homeostasis (37). Dysregulated expression of CTSH has been reported in various cancers, including breast cancer (38), prostate cancer (39), and glioma (40). Emerging evidence also links CTSH to regulation of inflammatory responses and cell death. For example, it modulates microglia-mediated inflammation and apoptosis following brain injury or infection (41). Additionally, CTSH is an important regulator of β-cell function in type 1 diabetes, where its overexpression protects against β-cell apoptosis, while downregulation promotes cell death (42). These findings suggest that CTSH may similarly influence inflammatory and cell death processes relevant to periodontal disease progression. AKR1B1 (Aldo-keto reductase family 1 member B1) is a key enzyme in the polyol pathway that contributes to oxidative stress and inflammatory signaling (43). It has been implicated in the pathogenesis of various inflammation-related conditions, including asthma (44), sepsis (45), and uveitis (46). AKR1B1 inhibition attenuates inflammatory signaling by downregulating lipopolysaccharide (LPS)-induced cascades and reducing the production of pro-inflammatory cytokines and chemokines (45, 47). These findings suggest that AKR1B1 may represent a promising therapeutic target in inflammatory diseases. IL24 (Interleukin 24) is a cytokine of the IL-10 family with known immunomodulatory and pro-apoptotic functions (48). It has been shown to induce apoptosis in various tumor cell types and modulate inflammatory signaling in immune cells (49). While its role in periodontal tissues remains unclear, elevated IL24 expression may reflect a heightened inflammatory state. From a diagnostic perspective, the robust and stable contribution of these genes to the machine learning model supports their potential as a molecular signature for PD. They hold promise for early detection, risk stratification, and longitudinal disease monitoring, and may improve diagnostic specificity by distinguishing PD from other inflammatory oral conditions. Integration of these markers into targeted PCR panels or transcriptomic assays could advance precision dentistry approaches in PD management.

Several limitations of this study should be noted. First, although single-cell RNA sequencing and spatial transcriptomics offered valuable insights into the heterogeneity and spatial patterns of PANoptosis activity in HGFs, the relatively small sample size may limit the broader applicability of our findings. Second, the identification of PANoptosis relied mainly on transcriptomic signatures rather than direct functional or protein-level assays, which may not fully capture the dynamic regulation and execution of this cell death pathway. Third, pseudotime trajectory analysis revealed a gradual transcriptional transition from LP-HGFs to HP-HGFs, indicating a potential phenotypic shift. However, this inference is based solely on transcriptional dynamics and does not constitute direct evidence of a lineage transition. Therefore, we propose this as a hypothetical differentiation model, which requires further validation through functional assays. Fourth, the consensus clustering-based molecular subtyping of PD was performed using a single publicly available bulk RNA-seq dataset (GSE16134). Although internal validation supported the robustness of this classification, the lack of multi-cohort external validation may limit the generalizability of the identified subtypes. Future studies incorporating additional independent datasets will be essential to confirm the reproducibility and stability of these PANoptosis-related subtypes. Moreover, although the single-cell and spatial transcriptomic results suggest a potential association between PANoptosis and immune modulation, these findings are based on correlative analyses. Further *in vitro* and *in vivo* experiments are needed to investigate and validate the underlying mechanisms linking PANoptosis to immune cell recruitment and activation. Finally, this study primarily focused on HGFs; thus, the involvement of other stromal or immune cell populations in PANoptosis-related PD pathogenesis remains to be explored.

## 5 Conclusion

In summary, this study reveals novel insights into PANoptosis in PD pathogenesis. By integrating single-cell RNA sequencing, spatial transcriptomics, and cell-cell communication analyses, we demonstrated that PANoptosis is markedly activated in HGFs from PD tissues and is associated with enhanced immune interactions. PANoptotic HGFs not only undergo inflammatory programmed cell death but also actively participate in shaping the immune microenvironment, potentially contributing to persistent inflammation and tissue destruction. Moreover, through screening 113 machine learning models, we identified four key PRGs and confirmed their expression in gingival tissues from PD patients. These findings reveal PANoptosis as an insufficiently recognized mechanism in periodontal disease and suggest that targeting PANoptosis pathways and hub genes could provide novel therapeutic avenues for controlling inflammation and maintaining periodontal tissue health.

## Data Availability

The original contributions presented in the study are included in the article/**Supplementary Material**. Further inquiries can be directed to the corresponding authors.

## Supplementary material

9The Supplementary Material for this article can be found online at: https://www.frontiersin.org/articles/10.3389/fimmu.2025.1671919/full#supplementary-material

## References

[1] Heitz-Mayfield LJA. Conventional diagnostic criteria for periodontal diseases (plaque-induced gingivitis and periodontitis). Periodontol 2000. (2024) 95:10–9. doi: 10.1111/prd.12579, PMID: 38831568

[2] Neurath N Kesting M. Cytokines in gingivitis and periodontitis: from pathogenesis to therapeutic targets. Front Immunol. (2024) 15:1435054. doi: 10.3389/fimmu.2024.1435054, PMID: 39253090 PMC11381234

[3] Song B Zhou T Yang WL Liu J Shao LQ. Programmed cell death in periodontitis: recent advances and future perspectives. Oral Dis. (2017) 23:609–19. doi: 10.1111/odi.12574, PMID: 27576069

[4] Xu X Zhang T Xia X Yin Y Yang S Ai D. Pyroptosis in periodontitis: From the intricate interaction with apoptosis, NETosis, and necroptosis to the therapeutic prospects. Front Cell Infect Microbiol. (2022) 12:953277. doi: 10.3389/fcimb.2022.953277, PMID: 36093182 PMC9450806

[5] Sordi MB Magini RS Panahipour L Gruber R. Pyroptosis-mediated periodontal disease. Int J Mol Sci. (2021) 23:372. doi: 10.3390/ijms23010372, PMID: 35008798 PMC8745163

[6] Malireddi RKS Kesavardhana S Kanneganti TD. ZBP1 and TAK1: master regulators of NLRP3 inflammasome/pyroptosis, apoptosis, and necroptosis (PAN-optosis). Front Cell Infect Microbiol. (2019) 9:406. doi: 10.3389/fcimb.2019.00406, PMID: 31850239 PMC6902032

[7] Pandeya A Kanneganti TD. Therapeutic potential of PANoptosis: innate sensors, inflammasomes, and RIPKs in PANoptosomes. Trends Mol Med. (2024) 30:74–88. doi: 10.1016/j.molmed.2023.10.001, PMID: 37977994 PMC10842719

[8] Sun X Yang Y Meng X Li J Liu X Liu H. PANoptosis: Mechanisms, biology, and role in disease. Immunol Rev. (2024) 321:246–62. doi: 10.1111/imr.13279, PMID: 37823450

[9] Zhu P Ke ZR Chen JX Li SJ Ma TL Fan XL. Advances in mechanism and regulation of PANoptosis: Prospects in disease treatment. Front Immunol. (2023) 14:1120034. doi: 10.3389/fimmu.2023.1120034, PMID: 36845112 PMC9948402

[10] Wu Q Qi S Kang Z Bai X Li Z Cheng J. PANoptosis in sepsis: A central role and emerging therapeutic target. J Inflammation Res. (2025) 18:6245–61. doi: 10.2147/jir.S513367, PMID: 40386177 PMC12085136

[11] Liu K Wang M Li D Duc Duong NT Liu Y Ma J. PANoptosis in autoimmune diseases interplay between apoptosis, necrosis, and pyroptosis. Front Immunol. (2024) 15:1502855. doi: 10.3389/fimmu.2024.1502855, PMID: 39544942 PMC11560468

[12] Gong W Liu Z Wang Y Huang W Yang K Gao Z. Reprogramming of Treg cell-derived small extracellular vesicles effectively prevents intestinal inflammation from PANoptosis by blocking mitochondrial oxidative stress. Trends Biotechnol. (2025) 43:893–917. doi: 10.1016/j.tibtech.2024.11.017, PMID: 39689981

[13] Chen S Jiang J Li T Huang L. PANoptosis: mechanism and role in pulmonary diseases. Int J Mol Sci. (2023) 24:5343. doi: 10.3390/ijms242015343, PMID: 37895022 PMC10607352

[14] Zhang A Zhang C Zhang Y Hu T Cheng R. PANoptosis is a compound death in periodontitis: A systematic review of ex vivo and *in vivo* studies. Oral Dis. (2024) 30:1828–42. doi: 10.1111/odi.14726, PMID: 37650218

[15] Pan S Li Y He H Cheng S Li J Pathak JL. Identification of ferroptosis, necroptosis, and pyroptosis-associated genes in periodontitis-affected human periodontal tissue using integrated bioinformatic analysis. Front Pharmacol. (2022) 13:1098851. doi: 10.3389/fphar.2022.1098851, PMID: 36686646 PMC9852864

[16] Yue Y Chan W Zhang J Liu J Wang M Hao L. Activation of receptor-interacting protein 3-mediated necroptosis accelerates periodontitis in mice. Oral Dis. (2024) 30:2485–96. doi: 10.1111/odi.14693, PMID: 37518945

[17] Jiang W Deng Z Dai X Zhao W. PANoptosis: A new insight into oral infectious diseases. Front Immunol. (2021) 12:789610. doi: 10.3389/fimmu.2021.789610, PMID: 34970269 PMC8712492

[18] Wielento A Lagosz-Cwik KB Potempa J Grabiec AM. The role of gingival fibroblasts in the pathogenesis of periodontitis. J Dent Res. (2023) 102:489–96. doi: 10.1177/00220345231151921, PMID: 36883660 PMC10249005

[19] Oka S Li X Sato F Zhang F Tewari N Kim IS. A deficiency of Dec2 triggers periodontal inflammation and pyroptosis. J Periodontal Res. (2021) 56:492–500. doi: 10.1111/jre.12849, PMID: 33641180

[20] Zhang K Chen X Zhou R Chen Z Wu B Qiu W. Inhibition of gingival fibroblast necroptosis mediated by RIPK3/MLKL attenuates periodontitis. J Clin Periodontol. (2023) 50:1264–79. doi: 10.1111/jcpe.13841, PMID: 37366309

[21] Zhang Y Guo Y Wei W Zhang Z Xu X. Metabolomics profiling reveals berberine-inhibited inflammatory response in human gingival fibroblasts by regulating the LPS-induced apoptosis signaling pathway. Front Pharmacol. (2022) 13:940224. doi: 10.3389/fphar.2022.940224, PMID: 36071855 PMC9441553

[22] Jin S Guerrero-Juarez CF Zhang L Chang I Ramos R Kuan CH. Inference and analysis of cell-cell communication using CellChat. Nat Commun. (2021) 12:1088. doi: 10.1038/s41467-021-21246-9, PMID: 33597522 PMC7889871

[23] Langfelder P Horvath S. WGCNA: an R package for weighted correlation network analysis. BMC Bioinf. (2008) 9:559. doi: 10.1186/1471-2105-9-559, PMID: 19114008 PMC2631488

[24] Wu E. Yin X. Liang F. Zhou X. Hu J. Yuan W.. Analysis of immunogenic cell death in periodontitis based on scRNA-seq and bulk RNA-seq data. Front Immunol. (2024) 15:1438998. doi: 10.3389/fimmu.2024.1438998, PMID: 39555084 PMC11568468

[25] Wu E Gu F Zhuo Q Gao Z Zhang Y Li J. Exploration the role of pro-inflammatory fibroblasts and related markers in periodontitis: combing with scRNA-seq and bulk-seq data. Front Immunol. (2025) 16:1537046. doi: 10.3389/fimmu.2025.1537046, PMID: 40370461 PMC12074970

[26] Hajishengallis G. Periodontitis: from microbial immune subversion to systemic inflammation. Nat Rev Immunol. (2015) 15:30–44. doi: 10.1038/nri3785, PMID: 25534621 PMC4276050

[27] Wang PL Shirasu S Shinohara M Daito M Oido M Kowashi Y. Induction of apoptosis in human gingival fibroblasts by a Porphyromonas gingivalis protease preparation. Arch Oral Biol. (1999) 44:337–42. doi: 10.1016/s0003-9969(99)00002-3, PMID: 10348360

[28] Xiang X Zhang J Yue Y. Pyroptosis: A major trigger of excessive immune response in the gingiva. Oral Dis. (2024) 30:4152–60. doi: 10.1111/odi.15013, PMID: 38852159

[29] Karki R Sharma BR Tuladhar S Williams EP Zalduondo L Samir P. Synergism of TNF-α and IFN-γ Triggers inflammatory cell death, tissue damage, and mortality in SARS-CoV-2 infection and cytokine shock syndromes. Cell. (2021) 184:149–168.e17. doi: 10.1016/j.cell.2020.11.025, PMID: 33278357 PMC7674074

[30] Pandian N Kanneganti TD. PANoptosis: A unique innate immune inflammatory cell death modality. J Immunol. (2022) 209:1625–33. doi: 10.4049/jimmunol.2200508, PMID: 36253067 PMC9586465

[31] Malireddi RKS Karki R Sundaram B Kancharana B Lee S Samir P. Inflammatory cell death, PANoptosis, mediated by cytokines in diverse cancer lineages inhibits tumor growth. Immunohorizons. (2021) 5:568–80. doi: 10.4049/immunohorizons.2100059, PMID: 34290111 PMC8522052

[32] Zhou J Windsor LJ. Porphyromonas gingivalis affects host collagen degradation by affecting expression, activation, and inhibition of matrix metalloproteinases. J Periodontal Res. (2006) 41:47–54. doi: 10.1111/j.1600-0765.2005.00835.x, PMID: 16409255

[33] Kim SH Jung IR Hwang SS. Emerging role of anti-proliferative protein BTG1 and BTG2. BMB Rep. (2022) 55:380–8. doi: 10.5483/BMBRep.2022.55.8.092, PMID: 35880434 PMC9442347

[34] Rouault JP Falette N Guéhenneux F Guillot C Rimokh R Wang Q. Identification of BTG2, an antiproliferative p53-dependent component of the DNA damage cellular response pathway. Nat Genet. (1996) 14:482–6. doi: 10.1038/ng1296-482, PMID: 8944033

[35] Imran M Lim IK. Regulation of Btg2(/TIS21/PC3) expression via reactive oxygen species-protein kinase C-NFκB pathway under stress conditions. Cell Signal. (2013) 25:2400–12. doi: 10.1016/j.cellsig.2013.07.015, PMID: 23876794

[36] Chiang KC Tsui KH Chung LC Yeh CN Feng TH Chen WT. Cisplatin modulates B-cell translocation gene 2 to attenuate cell proliferation of prostate carcinoma cells in both p53-dependent and p53-independent pathways. Sci Rep. (2014) 4:5511. doi: 10.1038/srep05511, PMID: 24981574 PMC4076686

[37] Wang Y Zhao J Gu Y Wang H Jiang M Zhao S. Cathepsin H: Molecular characteristics and clues to function and mechanism. Biochem Pharmacol. (2023) 212:115585. doi: 10.1016/j.bcp.2023.115585, PMID: 37148981

[38] Gabrijelcic D Svetic B Spaić D Skrk J Budihna M Dolenc I. Cathepsins B, H and L in human breast carcinoma. Eur J Clin Chem Clin Biochem. (1992) 30:69–74. PMID: 1316176

[39] Jevnikar Z Rojnik M Jamnik P Doljak B Fonovic UP Kos J. Cathepsin H mediates the processing of talin and regulates migration of prostate cancer cells. J Biol Chem. (2013) 288:2201–9. doi: 10.1074/jbc.M112.436394, PMID: 23204516 PMC3554893

[40] Sivaparvathi M Sawaya R Gokaslan ZL Chintala SK Rao JS. Expression and the role of cathepsin H in human glioma progression and invasion. Cancer Lett. (1996) 104:121–6. doi: 10.1016/0304-3835(96)04242-5, PMID: 8640738

[41] Fan K Li D Zhang Y Han C Liang J Hou C. The induction of neuronal death by up-regulated microglial cathepsin H in LPS-induced neuroinflammation. J Neuroinflamm. (2015) 12:54. doi: 10.1186/s12974-015-0268-x, PMID: 25889123 PMC4379721

[42] Fløyel T Brorsson C Nielsen LB Miani M Bang-Berthelsen CH Friedrichsen M. CTSH regulates β-cell function and disease progression in newly diagnosed type 1 diabetes patients. Proc Natl Acad Sci U S A. (2014) 111:10305–10. doi: 10.1073/pnas.1402571111, PMID: 24982147 PMC4104872

[43] Srivastava SK Yadav UC Reddy AB Saxena A Tammali R Shoeb M. Aldose reductase inhibition suppresses oxidative stress-induced inflammatory disorders. Chem Biol Interact. (2011) 191:330–8. doi: 10.1016/j.cbi.2011.02.023, PMID: 21354119 PMC3103634

[44] Yadav UC Ramana KV Aguilera-Aguirre L Boldogh I Boulares HA Srivastava SK. Inhibition of aldose reductase prevents experimental allergic airway inflammation in mice. PloS One. (2009) 4:e6535. doi: 10.1371/journal.pone.0006535, PMID: 19657391 PMC2717330

[45] Ramana KV Fadl AA Tammali R Reddy AB Chopra AK Srivastava SK. Aldose reductase mediates the lipopolysaccharide-induced release of inflammatory mediators in RAW264.7 murine macrophages. J Biol Chem. (2006) 281:33019–29. doi: 10.1074/jbc.M603819200, PMID: 16956889

[46] Yadav UC Srivastava SK Ramana KV. Aldose reductase inhibition prevents endotoxin-induced uveitis in rats. Invest Ophthalmol Vis Sci. (2007) 48:4634–42. doi: 10.1167/iovs.07-0485, PMID: 17898287 PMC2377062

[47] Reddy AB Srivastava SK Ramana KV. Anti-inflammatory effect of aldose reductase inhibition in murine polymicrobial sepsis. Cytokine. (2009) 48:170–6. doi: 10.1016/j.cyto.2009.07.004, PMID: 19660963 PMC2767443

[48] Smith S Lopez S Kim A Kasteri J Olumuyide E Punu K. Interleukin 24: Signal Transduction Pathways. Cancers (Basel). (2023) 15. doi: 10.3390/cancers15133365, PMID: 37444474 PMC10340555

[49] Gupta P Su ZZ Lebedeva IV Sarkar D Sauane M Emdad L. mda-7/IL-24: multifunctional cancer-specific apoptosis-inducing cytokine. Pharmacol Ther. (2006) 111:596–628. doi: 10.1016/j.pharmthera.2005.11.005, PMID: 16464504 PMC1781515

