# Supplementary material for: Single-cell and bulk transcriptomic analyses reveal PANoptosis-associated immune dysregulation of fibroblasts in periodontitis
Source: Front Immunol. 2025 Sep 5;16:1671919. doi: 10.3389/fimmu.2025.1671919 (PMC12446042; doi:10.3389/fimmu.2025.1671919)
Supplement: Supplementary file 1 [file SupplementaryFile1.zip › Suppl. Figure 6.docx]

Supplementary Material


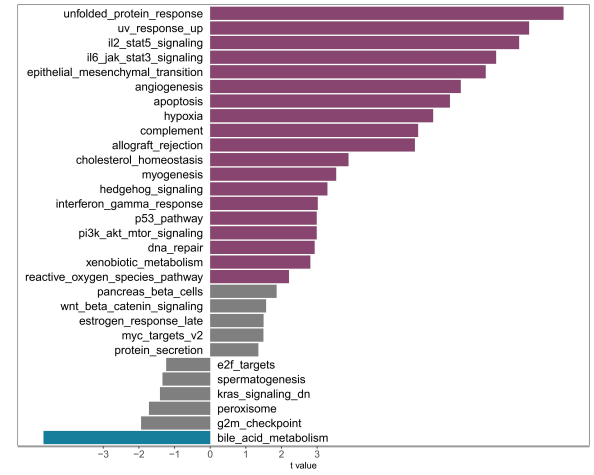

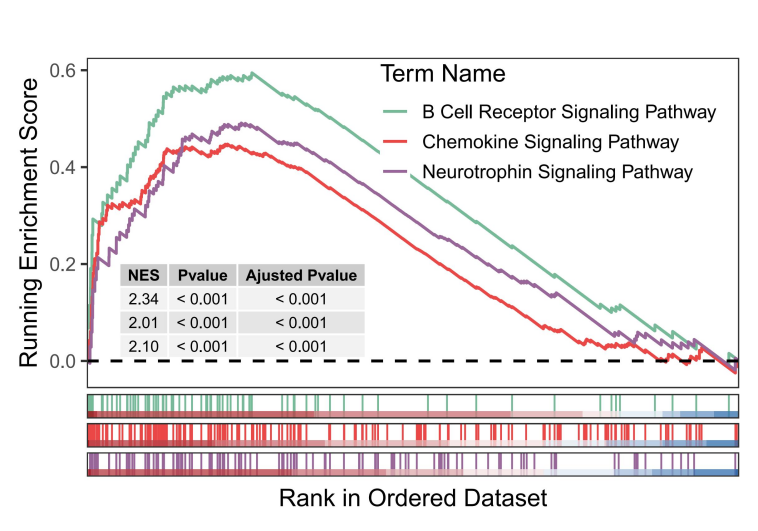


BTG2

BTG2

E

A

CTSH

CTSH

F

B


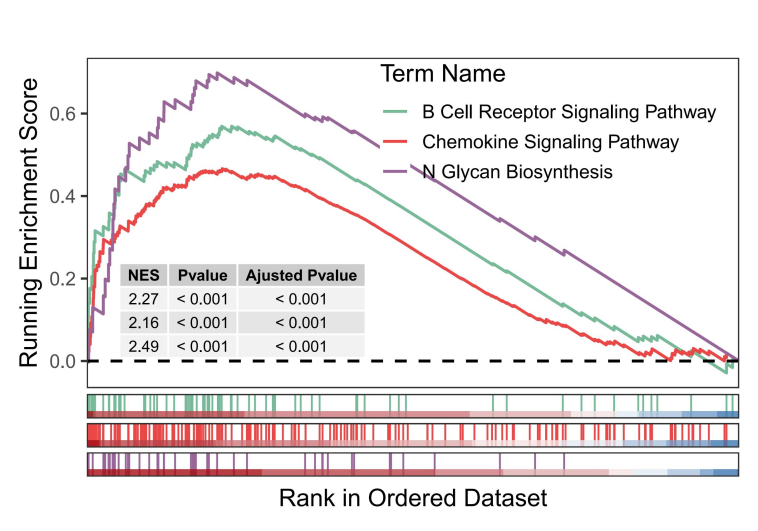


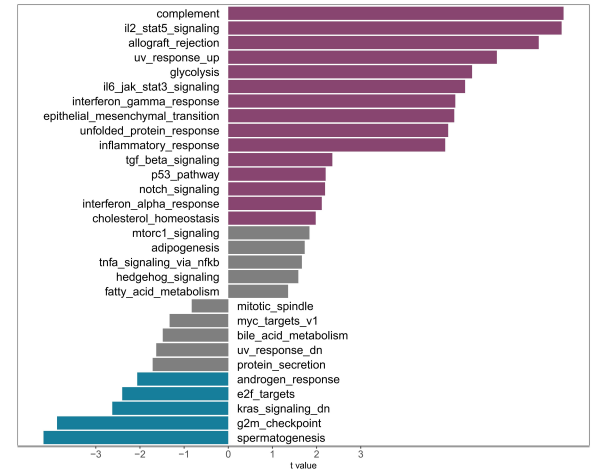


C


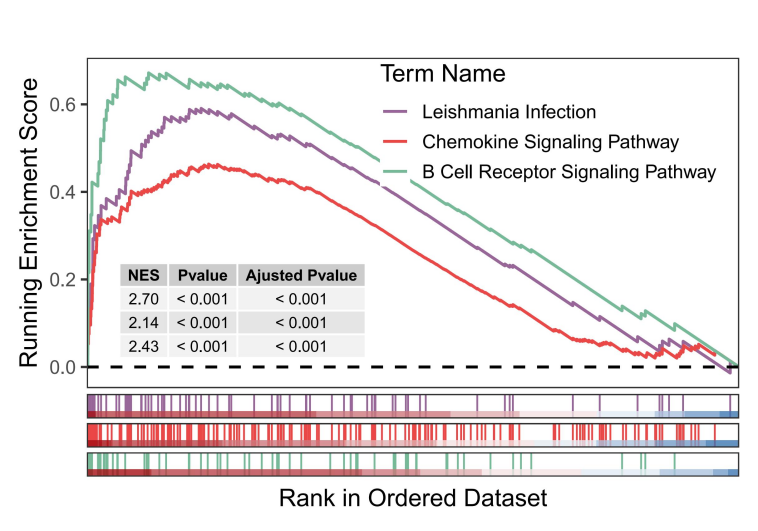


AKR1B1

AKR1B1

G


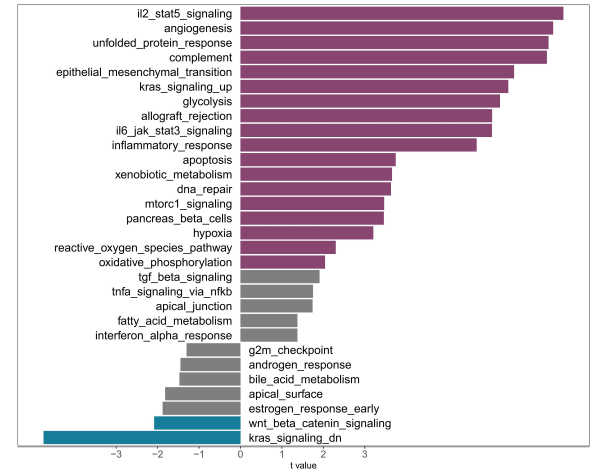


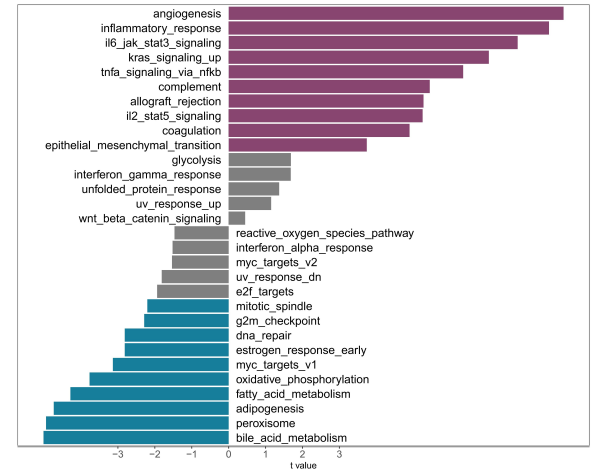

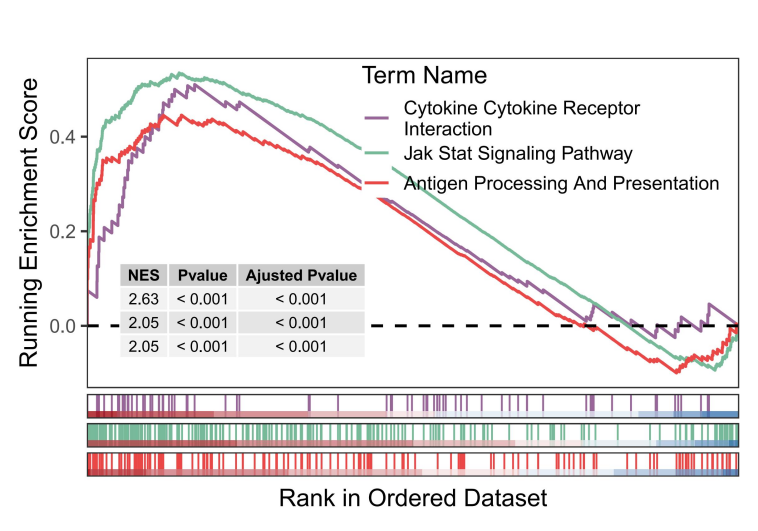


IL24

IL24

H

D

**Supplementary Figure 6**. Enrichment analysis for BTG2, CTSH, AKR1B1, and IL24 using GSVA and GSEA. (A-D) GSVA results showing differences in pathway activity between high- and low-expression groups of BTG2 (A), CTSH (B), AKR1B1 (C), and IL24 (D). The X-axis represents GSVA scores ranked in descending order, illustrating the relative enrichment of pathways. Purple and light blue lines indicate significantly enriched pathways, while gray lines represent non-significant ones. (E-H) GSEA results for BTG2 (E), CTSH (F), AKR1B1 (G), and IL24 (H), identifying pathways significantly associated with the upregulation or downregulation of each gene. NES, normalized enrichment score.
