# Supplementary material for: Single-cell and bulk transcriptomic analyses reveal PANoptosis-associated immune dysregulation of fibroblasts in periodontitis
Source: Front Immunol. 2025 Sep 5;16:1671919. doi: 10.3389/fimmu.2025.1671919 (PMC12446042; doi:10.3389/fimmu.2025.1671919)
Supplement: Supplementary file 1 [file SupplementaryFile1.zip › Suppl. Table 5.DOCX]

Supplementary Material

**Supplementary Table 5.** Sequences of the primers used for RT-qPCR.

| **Gene** | **Forward 5’-3’** | **Reverse 5’-3’** |
| --- | --- | --- |
| GAPDH | CTTTGGTATCGTGGAAGGACTC | GTAGAGGCAGGGATGATGTTCT |
| ZBP1 | GCAAACTCCGAAGCCATCCAGA | CCAAGTTGAGGAATCACCTGGTG |
| Caspase-1 | ACAGGCATGACAATGCTGCT | GCTGTCAGAGGTCTTGTGCT |
| Caspase-3 | GGAAGCGAATCAATGGACTCTGG | GCATCGACATCTGTACCAGACC |
| RIPK3 | ATGCTGCTGTCTCCACGGTAA | AAAGCCATCCATTTCTGTCCCTC |
| BTG2 | ACCTGCAAGAACCAAGTGCT | AGGTATGTGGTGGCCTGTTG |
| CTSH | TTGTGAAAAACTCTTGGGGCTC | TGAGGAATGGGATAGGAGGCA |
| AKR1B1 | GTCGGCAGATTGATGACATACTC | TGGCATTCCACCTGCAAGA |
| IL24 | GCCTCTGGATGCTGTGAAGA | ACTAAGCACCCATCCACTGC |
