# Supplementary material for: Single-cell and bulk transcriptomic analyses reveal PANoptosis-associated immune dysregulation of fibroblasts in periodontitis
Source: Front Immunol. 2025 Sep 5;16:1671919. doi: 10.3389/fimmu.2025.1671919 (PMC12446042; doi:10.3389/fimmu.2025.1671919)
Supplement: Supplementary file 1 [file SupplementaryFile1.zip › Suppl. Figure 4.DOCX]

Supplementary Material


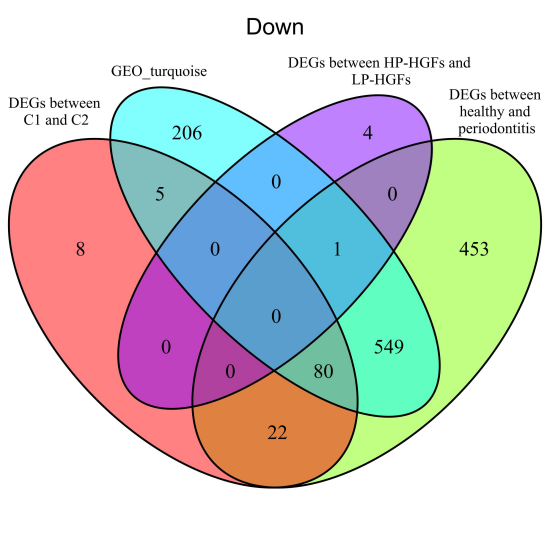

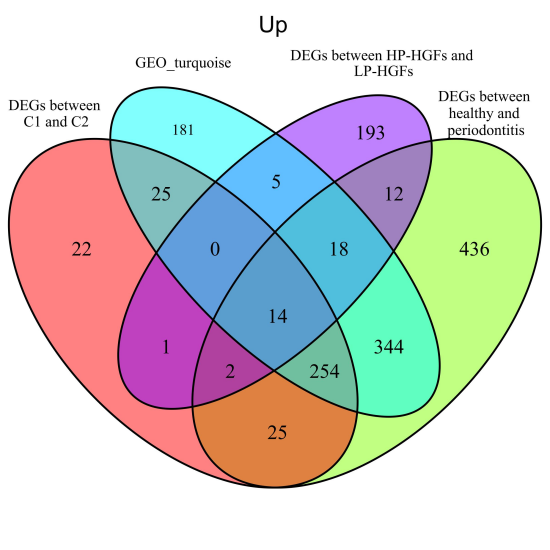


A

B

**Supplementary Figure 4.** (A-B) Venn diagram of upregulated and downregulated genes obtained by intersecting the WGCNA module, DEGs between healthy and periodontitis tissues, DEGs between C1 and C2 PANoptosis subgroups, and DEGs between HP-HGFs and LP-HGFs. DEG: Differentially expressed genes; HP-HGF: high-PANoptosis HGFs; LP-HGFs: low-PANoptosis HGFs.
