# Supplementary material for: Single-cell and bulk transcriptomic analyses reveal PANoptosis-associated immune dysregulation of fibroblasts in periodontitis
Source: Front Immunol. 2025 Sep 5;16:1671919. doi: 10.3389/fimmu.2025.1671919 (PMC12446042; doi:10.3389/fimmu.2025.1671919)
Supplement: Supplementary file 1 [file SupplementaryFile1.zip › Suppl. Figure 3.DOCX]

Supplementary Material


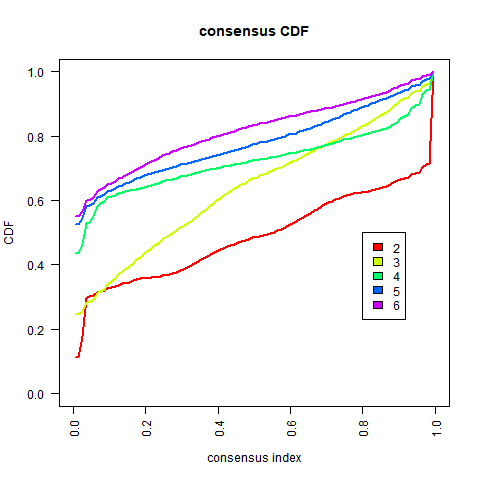


**Supplementary Figure 3**. Consensus cumulative distribution function (CDF) plot for determining the optimal number of clusters.
