# Supplementary material for: Single-cell and bulk transcriptomic analyses reveal PANoptosis-associated immune dysregulation of fibroblasts in periodontitis
Source: Front Immunol. 2025 Sep 5;16:1671919. doi: 10.3389/fimmu.2025.1671919 (PMC12446042; doi:10.3389/fimmu.2025.1671919)
Supplement: Supplementary file 1 [file SupplementaryFile1.zip › Suppl. Figure 2.DOCX]

Supplementary Material


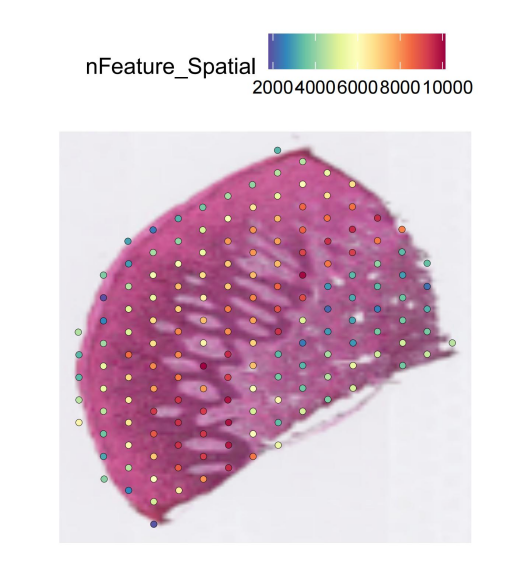

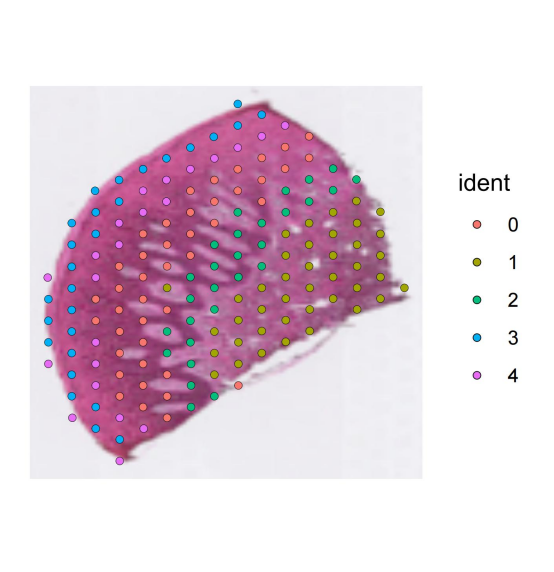

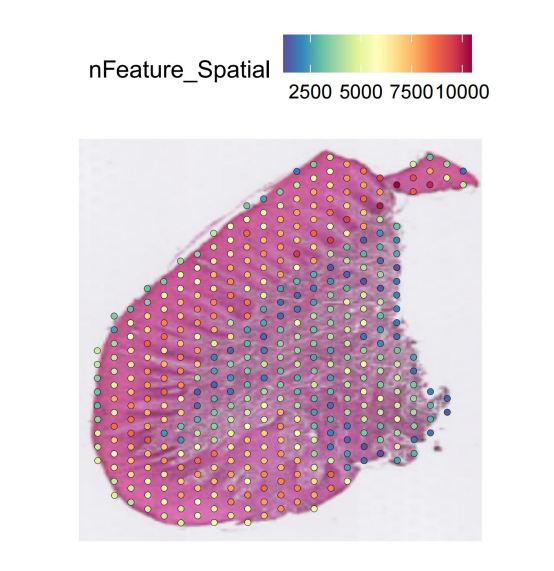

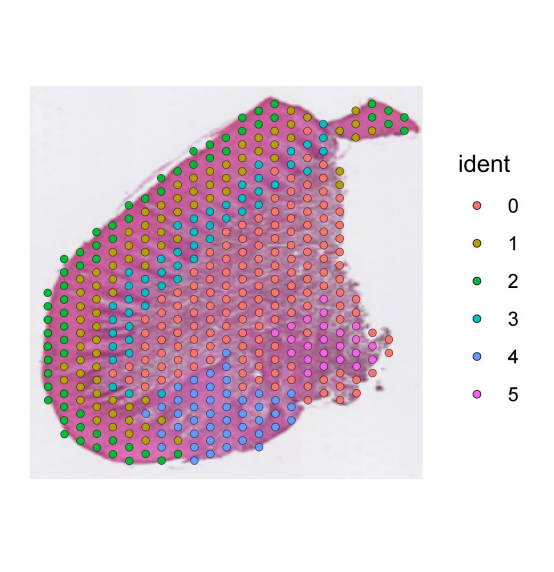


GSM6258256

GSM6258257

B

A

C

D

**Supplementary Figure 2.** Spatial distribution of detected genes and identified regions across tissue sections. (A-B) Spatial transcriptomics plots showing the number of detected genes (nFeature_Spatial) at each capture spot on the tissue sections. The color scale represents gene count per spot, ranging from low (blue) to high (red). (C-D) Spatial domain analysis identified six and five distinct regions, respectively, in the corresponding tissue slices.
