# Supplementary material for: Single-cell and bulk transcriptomic analyses reveal PANoptosis-associated immune dysregulation of fibroblasts in periodontitis
Source: Front Immunol. 2025 Sep 5;16:1671919. doi: 10.3389/fimmu.2025.1671919 (PMC12446042; doi:10.3389/fimmu.2025.1671919)
Supplement: Supplementary file 1 [file SupplementaryFile1.zip › Suppl. Figure 1.DOCX]

Supplementary Material


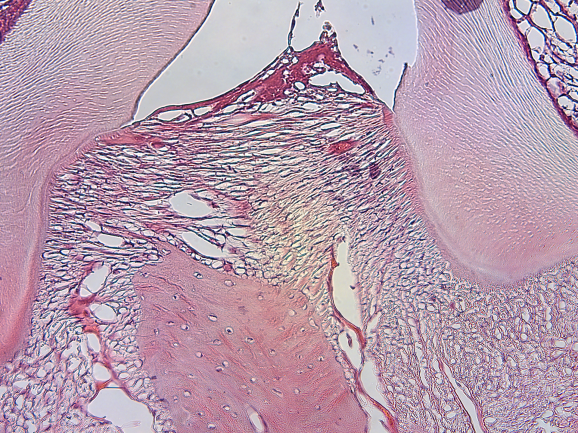

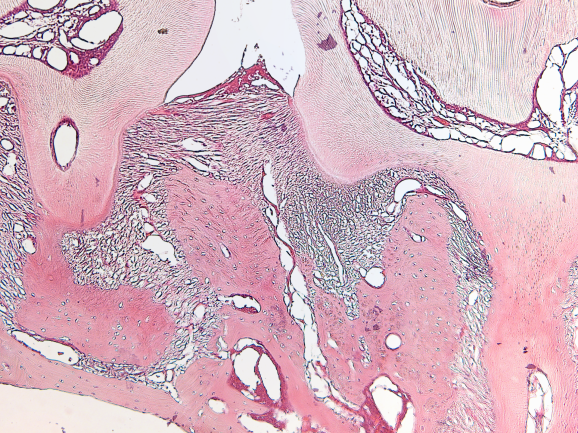

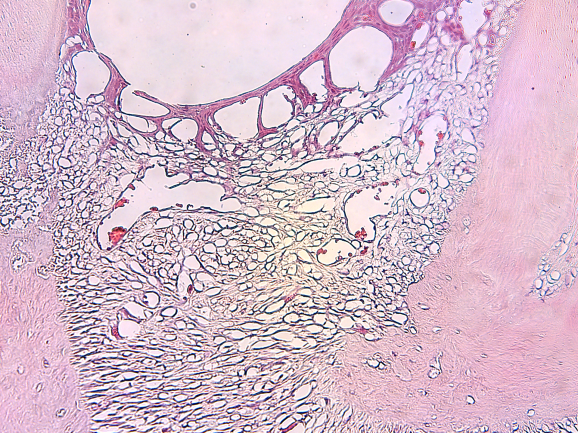

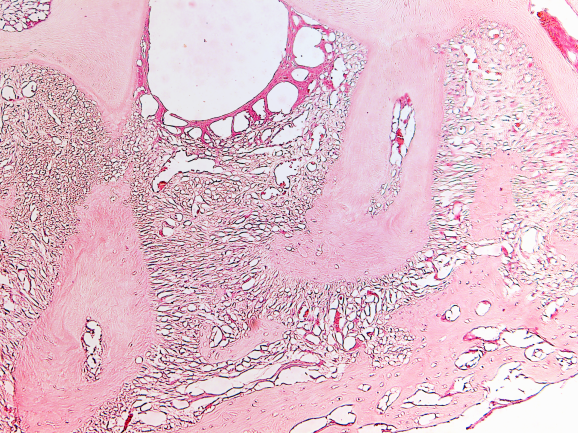


WT

PD

20X

40X

**Supplementary Figure 1**. Representative H&E-stained images of periodontal paraffin sections.
